# Supplementary material for: Chemical and Electrochemical Reductions of Monoiminoacenaphthenes
Source: Int J Mol Sci. 2023 May 12;24(10):8667. doi: 10.3390/ijms24108667 (PMC10218263; doi:10.3390/ijms24108667)
Supplement: Supplementary file 1 [file ijms-24-08667-s001.zip › ijms-2374208-supplementary.pdf]

International Journal of *Molecular Sciences*

MDPI

Supplementary

Differences between chemical and electrochemical reductions of monoiminoacenaphthenes

Vera V. Khrizanforova [1], Robert R. Fayzullin [1], Tatiana P. Gerasimova [1], Mikhail N. Khrizanforov [1], Almaz A. Zagidullin [1*], Daut R. Islamov [1], Anton N. Lukoyanov [2], Yulia H. Budnikova [1]

[1] Arbuzov Institute of Organic and Physical Chemistry, FRC Kazan Scientific Center, Russian Academy of Sciences, 8 Arbuzov Street, 420088 Kazan, Russia; khrizanforovavera@yandex.ru (V.V.K.); robert.fayzullin@gmail.com (R.R.F.); tatyanagr@gmail.com (T.P.G.); khrizanforov@gmail.com (M.N.K.); daut1989@mail.ru (D.R.I.); yulia@iopc.ru (Y.H.B.)
[2] A.M. Butlerov Institute of Chemistry, Kazan Federal University, 18 Kremlevskaya Street, 4 20008 Kazan, Russia
[3] G.A. Razuvaev Institute of Organometallic Chemistry, Russian Academy of Sciences, 49 Tropinin Street, 603137 Nizhny Novgorod, Russia; anton_lukoyanov@mail.ru
* Correspondence: zagidullin@iopc.ru

General information: S1-S3
Computational details: S3
Single Crystal X-ray Diffraction: S4-S10
NMR spectra: S10-S16
IR spectra: S17-S24
Calculated frontiers orbital view: S24-S25
Cyclic and difference-pulse voltammograms: S26-S29
Experimental and predicted UV/Vis spectra: S30-S32

General information. All manipulations were carried out in vacuo or under nitrogen using the standard Schlenk technique or in a glovebox. The solvents (THF, toluene, diethyl ether, and hexane) were distilled from sodium/benzophenone and stored over 3 Å molecular sieves under nitrogen. MeCN with a purity 99.99 % were stored under 3 Å molecular sieves or a septum in a glovebox atmosphere and used without additional purification. Acenaphtenequinone, primary amines, sodium metal, $NaBH_4$, and Bu_4NPF_6 were purchased and used without any preliminary purification.

Synthesis of MIANs I-IX. The MIANs **I-IX** was synthesized according to the published procedure [1]. A mixture of acenaphthenequinone (11 mmol), corresponding primary amine (11 mmol), and formic acid (2 mmol) was refluxed in toluene (50 mL) for 2 h. After cooling to ambient temperature the solution was filtered. All volatiles were removed from the filtrate under vacuum and the residue was purified using column chromatography with DCM as eluent. The total yield of MIANs was about 70%.

I: ^{1}H NMR (200 MHz: $CDCl_3$) δ 8.18 (2H, d, J=7.6 Hz), 8.01 (1H, d, J=6.4 Hz), 7.83 (1H, d, J=7.6 Hz), 7.45 (3H, q, J=7.6 Hz), 7.27 (1H, d, J=5.2 Hz), 7.09 (2H, d, J=7.4 Hz), 6.97 (1H, d, J=7.2 Hz). ESI MS (m/z): found: 258.10; calc.: 258.09. IR (KBr, cm^{-1}): 1891, 1879, 1825, 1735, 1719, 1651, 1602, 1592, 1483, 1454, 1435, 1421, 1359, 1347.

II: ^{1}H NMR (200 MHz: $CDCl_3$) δ 8.17 (2H, d, J=7.8 Hz), 8.00 (1H, d, J=8.4 Hz), 7.80 (1H, t, J=7.8 Hz), 7.45 (1H, t, J=8.0 Hz), 7.25 (2H, s), 7.11 (1H, d, J=7.2 Hz), 7.01 (2H, d, J=8.0 Hz), 2.44 (3H, s). ESI MS (m/z): found: 272.12; calc.: 272.10 IR (KBr, cm^{-1}): 1942, 1881, 1828, 1731, 1656, 1602, 1590, 1502, 1489, 1453, 1435, 1421, 1378, 1358, 1308.

III: ^{1}H NMR (200 MHz: $CDCl_3$) δ 8.15 (2H, m), 8.00 (1H, d, J=8.2 Hz), 7.78 (1H, m), 7.43 (1H, m), 7.26 (1H, m), 7.07 (6H, m), 3.88 (3H, s). ESI MS (m/z): found: 288.14; calc.: 288.10 IR (KBr, cm^{-1}): 1719, 1633, 1599, 1587, 1503, 1489, 1463, 1438, 1420, 1294.

IV: ^{1}H NMR (400 MHz: $CDCl_3$) δ 8.28 (1H, d, J=8.4 Hz), 8.19 (1H, d, J=8.0 Hz), 8.11 (1H, d, J=7.2 Hz), 8.01 (1H, d, J=8.4 Hz), 7.85 (1H, m), 7.44 (1H, t, J=7.2 Hz), 7.15 (1H, m), 7.11 (1H, m), 6.73 (1H, d, J=7.2

Hz), 2.06 (6H, s). ESI MS (m/z): found: 286.16; calc.: 286.12 IR (KBr, cm^{-1}): 1921, 1904, 1835, 1728, 1647, 1600, 1589, 1488, 1462, 1435, 1421, 1375, 1359.

V: ^{1}H NMR (400 MHz: $CDCl_3$) δ 8.17 (1H, d, J=8.0 Hz), 7.99 (1H, d, J=8.4 Hz), 7.80 (1H, t, J=7.6 Hz), 7.39 (1H, t, J=7.6 Hz), 6.64 (1H, d, J=7.2 Hz), 2.85 (2H, m), 1.16 (6H, d, J=6.8 Hz), 0.90 (6H, d, J=6.8 Hz). ESI MS (m/z): found: 342.28; calc.: 342.18. IR (KBr, cm^{-1}): 1981, 1919, 1852, 1730, 1658, 1602, 1590, 1488, 1465, 1433, 1419, 1382, 1360, 1328.

VI: ^{1}H NMR (200 MHz: $CDCl_3$) δ 8.17 (1H, d, J=7.6 Hz), 8.0 (1H, d, J=8.4 Hz), 7.81 (1H, t, J=7.6 Hz), 7.44 (1H, t, J=7.4 Hz), 6.95 (2H, m), 6.78 (1H, d, J=7.0 Hz), 2.36 (3H, s), 2.00 (6H, s). ESI MS (m/z): found: 300.19; calc.: 300.13. IR (KBr, cm^{-1}): 1723, 1649, 1601, 1590, 1474, 1431, 1419, 1373, 1276.

VII: ^{1}H NMR (200 MHz: $CDCl_3$) δ 8.20 (6H, m), 7.85 (6H, m). ESI MS (m/z): found: 308.18; calc.: 308.10. IR (KBr, cm^{-1}): 1974, 1772, 1719, 1644, 1602, 1591, 1517, 1488, 1459, 1437, 1419, 1389, 1307.

VIII: ^{1}H NMR (400 MHz: $CDCl_3$) δ 8.78 (2H, dd, J=1.6 Hz), 8.27 (1H, d, J=8.0 Hz), 8.10 (1H, d, J=8.0 Hz), 8.08 (1H, d, J=2.0 Hz), 7.85 (1H, m), 7.39 (1H, t, J=4.0 Hz), 7.37 (1H, d, J=3.2 Hz), 7.34 (1H, d, J=7.6 Hz), 7.18 (2H, dd, J=7.2 Hz), 6.95 (2H, dd, J=6.4 Hz). ESI MS (m/z): found: 309.12; calc.: 309.10. IR (KBr, cm^{-1}): 1922, 1709, 1623, 1597, 1583, 1570, 1496, 1465, 1447, 1433, 1417, 1384, 1322.

Synthesis of sodium complexes 1-9-Na

In a glovebox, 1 equiv. of ligand **I-IX** and 1.1 equiv. of metallic sodium were dissolved in THF and stirred for a few days at room temperature. After the violet solution was filtered and dried in a vacuum. Sodium complexes **1-9-Na** were extracted with diethyl ester. The yield was about 40-60%. The crystals suitable for XRD were obtained from THF/hexane solutions at –30 °C.

Electrochemical synthesis of **1-9**-NBu_4. *In situ* electrochemical reduction of THF solution of MIANs **I-IX** was carried out using a BASi Epsilon potentiostat in controlled-potential mode. 0.05 mmol of MIAN was dissolved in 0.1M solution of Bu_4NPF_6 in THF. The electrolysis potential was found from cyclic voltammetry studies performed earlier. The electrolysis potential was–1.9 - –2.0 V. Electrolysis was performed in a three-electrode divided cell. The surface area of the platinum cylindrical anode used as the working electrode was 10.0 cm2. A ceramic plate with a pore size of 10 μm was used as a membrane. A platinum grid served as a anode. During electrolysis, the electrolyte was stirred using a magnetic stirrer. The potential of the reaction was measured vs. the Ag/$AgNO_3$ system. Bu_4NPF_6 was used as a supporting electrolyte in both cathode and anode sides. 2 Q of electricity per 1 mol of MIAN was passed. During electrolysis the solution was observed changing color from yellow-orange to dark blue. After the required amount of electricity (2ē reduction, 2 F electricity) was passed, electrolysis was finished, and aliquots of reduced MIANs were studied in situ using UV/Vis spectroscopy. The THF solution of reduced MIANs was dried in a vacuum and the desired product was extracted by diethyl ester. After storing the diethyl ester solution of **1**-**9**-NBu_4 at -35°C, the crystals suitable for XRD study were formed. The yield of **1**-**9**-NBu_4 was about 30%.

Reduction of V by $NaBH_4$.

1 equiv. of **V** and 2 equiv. of $NaBH_4$ were dissolved in THF and stirred overnight. Then, the solution was filtered and stored at -35°C for one week. After that, the crystals suitable for XRD study were formed with a yield of 70%.

References

[1] Razborov, D.A.; Lukoyanov, A.N.; Baranov, E.; Fedushkin, I.L. Addition of phenylacetylene to a magnesium complex of monoiminoacenaphtheneone (dpp-mian). Dalton Trans. 2015, 44, 20532–20541.

NMR measurements were performed in the NMR department (A.E. Arbuzov Institute Organic and Physical Chemistry) of the Federal Collective Spectral Analysis Center for physical and chemical studies on the structure, properties, and composition of matter and materials. NMR experiments were conducted using Bruker spectrometers AVANCE-400 (399.93 MHz (1H), 100.6 MHz (13C) and AVANCE-600 (600.1 MHz (1H), 150.9 MHz (13C)) equipped with a pulsed gradient unit capable of producing magnetic field pulse gradients in the z-direction of 53.5 G cm-1. Chemical shifts are reported on the δ (ppm) scale relative to the residual solvent signals for 1H and 13C.

Electrochemical measurements. Cyclic voltammetry measurements were performed with an E2P potentiostat of BASi Epsilon (USA) composed of a measuring block, a Dell Optiplex 320 computer with installed an EpsilonES-USB-V200 program, and a C3 electrochemical cell. A stationary glassy-carbon electrode (with diameter of 3.0 mm) was used as a working electrode. Ferrocene was used as an internal standard. Ag/AgCl was used as a comparison electrode. Before and after each CV and CPE experiment, a 1.5 mM solution of ferrocene was recorded under similar conditions. The potential was reproduced and remained constant in all the cases. A platinum wire of 0.5 mm diameter was used as an auxiliary electrode. Measurements were performed under the inert nitrogen atmosphere of a glovebox.

EPR spectroscopy. EPR spectra were registered on a Bruker ELEXSYS E500 Xrange spectrometer. The solution or powder of samples were prepared and placed in a quartz calcined ampule in a glove box inert atmosphere and then soldered.

UV/Vis spectroscopy. UV/Vis spectra were recorded at room temperature on a PerkinElmer Lambda 365 spectrometer. Spectra were registered with a scan speed of 480 nm min−1, using a spectral width of 1 nm with 10 mm quartz cuvettes. A thin layer quartz glass spectroelectrochemical cell with a Pt counter electrode and a Pt gauze working electrode were used for UV/Vis spectroelectrochemical experiments. A solution of sample was placed in a spectroelectrochemical UV/Vis quartz cuvette in a glove box and after that the reduced samples were obtained in situ in the spectroelectrochemical cell.

IR spectra of solid compounds were registered using Bruker Vector-27 FTIR spectrometer in the 400–4000 cm−1 range (optical resolution 4 cm−1). The samples were prepared as KBr pellets.

The ESI MS measurements were performed using an AmazonX ion trap mass spectrometer (Bruker Daltonic GmbH, Germany) in positive (and/or negative) mode in the mass range of 70–3000. The capillary voltage was −3500 V, nitrogen drying gas – 10 L•min^{-1}, dissolving temperature – 250 °C. A methanol/water solution (70:30) was used as a mobile phase at a flow rate of 0.2 mL/min with a binary pump (Agilent 1260 chromatograph, USA). The sample was dissolved in methanol to a concentration of 10-6 g•L^{-1}. The instrument was calibrated with a tuning mixture (Agilent G2431A, USA). For instrument control and data acquiring the TrapControl 7.0 software (Bruker Daltonik GmbH, Germany) was used. Data processing was performed by DataAnalysis 4.0 SP4 software (Bruker Daltonik GmbH, Germany).

Computational Methods. Quantum chemical calculations were performed with the Gaussian 16 [1] suite of programs. The ground state structures were optimized with the use of hybrid PBE0 functional [2] and the Ahlrichs' triple-ζ def-TZVP AO basis set [3]. In all geometry optimizations, the D3 approach [3] to describe the London dispersion interactions together with the Becke–Johnson damping function [4-6] were employed as implemented in the Gaussian 16 program. Time-Dependent Density Functional Response Theory (TDDFT) was employed to compute the vertical excitation energies (i.e., absorption wavelengths) and oscillator strengths for the ground-state optimized geometries in the gas phase, 50 lowest singlet excited states were taken into account. The optimization of ground all compounds as well as vertical shifts were calculated with the use of polarizable continuum model [7, 8].

[1] Frisch, M. J.; Trucks, G. W.; Schlegel, H. B.; Scuseria, G. E.; Robb, M. A.; Cheeseman, J. R.; Scalmani, G.; Barone, V.; Petersson, G. A.; Nakatsuji, H.; Li X.; Caricato M.; Marenich A.; Bloino J.; Janesko B. G.; Gomperts R.; Mennucci B.; Hratchian H. P.; Ortiz J. V.; Izmaylov A. F.; Sonnenberg J. L.; Williams-Young D.; Ding F.; Lipparini F.; Egidi F.; Goings J.; Peng B.; Petrone A.; Henderson T.; Ranasinghe D.; Zakrzewski V. G.; Gao J.; Rega N.; Zheng G.; Liang W.; Hada M.; Ehara M.; Toyota K.; Fukuda R.; Hasegawa J.; Ishida M.; Nakajima T.; Honda Y.; Kitao O.; Nakai H.; Vreven T.; Throssell K.; Montgomery J. A.; Peralta J. E.; Ogliaro F.; Bearpark M.; Heyd J. J.; Brothers E.; Kudin K. N.; Staroverov V. N.; Keith T.; Kobayashi R.; Normand J.; Raghavachari K.; Rendell A.; Burant J. C.; Iyengar S. S.; Tomasi J.; Cossi M.; Millam J. M.; Klene M.; Adamo C.; Cammi R.; Ochterski J. W.; Martin R. L.; Morokuma K.; Farkas O.; Foresman J. B.; Fox D. J. Gaussian 09 Revision A .02 Gaussian, Inc., *Wallingford CT*, **2016**. (53)

[2] Adamo, C.; Barone, V. Toward Reliable Density Functional Methods without Adjustable Parameters: The PBE0 Model. *J. Chem. Phys.* **1999**, *110* (13), 6158–6170. https://doi.org/10.1063/1.478522.

[3] Weigend, F.; Ahlrichs, R. Balanced Basis Sets of Split Valence, Triple Zeta Valence and Quadruple Zeta Valence Quality for H to Rn: Design and Assessment of Accuracy. *Phys. Chem. Chem. Phys.* **2005**, *7* (18), 3297. https://doi.org/10.1039/b508541a.

[4] Grimme, S.; Antony, J.; Ehrlich, S.; Krieg, H. A Consistent and Accurate Ab Initio Parametrization of Density Functional Dispersion Correction (DFT-D) for the 94 Elements H-Pu. *J. Chem. Phys.* **2010**, *132* (15), 154104-1-154104–154119. https://doi.org/10.1063/1.3382344.

[5] Johnson, E. R.; Becke, A. D. A Post-Hartree-Fock Model of Intermolecular Interactions: Inclusion of Higher-Order Corrections. *J. Chem. Phys.* **2006**, *124* (17), 174104-1-174104–174109. https://doi.org/10.1063/1.2190220.
[6] Grimme, S.; Ehrlich, S.; Goerigk, L. Effect of the Damping Function in Dispersion Corrected Density Functional Theory. *J. Comput. Chem.* **2011**, *32* (7), 1456–1465. https://doi.org/10.1002/jcc.21759.
[7] Becke, A. D.; Johnson, E. R. A Density-Functional Model of the Dispersion Interaction. *J. Chem. Phys.* **2005**, *123* (15), 154101-1-154101–154109. https://doi.org/10.1063/1.2065267.
[8] S. Miertuš, E. Scrocco, J. Tomasi, Electrostatic Interaction of a Solute with a Continuum. A Direct Utilization of ab initio Molecular Potentials for the Prevision of Solvent Effects, Chem. Phys. 1981; 55: 117-29. DOI: 10.1016/0301-0104(81)85090-2
[9] M. Cossi, G. Scalmani, N. Rega, V. Barone, New developments in the polarizable continuum model for quantum mechanical and classical calculations on molecules in solution, J. Chem. Phys. 2008; 117: 43-54. DOI: 10.1063/1.1480445

Single Crystal X-ray Diffraction

The X-ray diffraction (XRD) data for the single crystals of **3**-NBu_4, **6**-Na, **4**-NBu_4, and **7**-Na were collected on a Rigaku XtaLab Synergy S instrument with a HyPix detector and a PhotonJet microfocus X-ray tube using Cu *Kα* (1.54184 Å) radiation. Data sets for the single crystals of compounds **II**, **III**, **IX**, **1**-NBu_4, **5**-Na, and **5**-$NaBH_4$ were collected on a Bruker D8 QUEST diffractometer with a PHOTON III area detector and an IμS DIAMOND microfocus X-ray tube using Mo *Kα* (0.71073 Å) radiation. The diffractometers were equipped with cryo-systems for low temperature experiments. The data reduction packages *CrysAlisPro* or *APEX*4 were used for data processing. Analysis of the integrated data did not show any decay. Data were corrected for systematic errors and absorption: Numerical absorption correction based on integration over a multifaceted crystal model and empirical absorption correction based on spherical harmonics according to the point group symmetry using equivalent reflections. The structures were solved by the intrinsic phasing approach using *SHELXT*-2018/2[1] and refined by the full-matrix least-squares on F^2 using *SHELXL*-2018/3.[2] Calculations were mainly performed using *WinGX*-2021.3[3] and *Olex*2 v1.5.[4] Non-hydrogen atoms were refined anisotropically. Hydrogen atoms were inserted at the calculated positions and refined as riding atoms or found using a rotating group refinement with idealized tetrahedral angles. The disorder, if present, was refined using a free variable and reasonable restraints on geometry and anisotropic displacement parameters. The unit cell of **5**-Na contained highly disordered solvent molecules (THF and hexane), which were treated as a diffuse contribution to the overall scattering without specific atom positions. Squeezed solvent information is not included in the formulae, nor are related items such as molecular weights and calculated densities. The compounds studied have no unusual bond lengths and angles.

Deposition numbers CCDC 2253912-2253916, 2254012-2254015, and 2256110 contain the supplementary crystallographic data for this paper. These data are provided free of charge by the joint Cambridge Crystallographic Data Centre and Fachinformationszentrum Karlsruhe Access Structures service www.ccdc.cam.ac.uk/structures.

Crystallographic data for II.

$C_{19}H_{13}NO$, orange prism (0.282 × 0.172 × 0.126 mm³), formula weight 271.30 g mol⁻¹; monoclinic, $P2_1/m$ (No. 11), a = 9.0095(3) Å, b = 6.8258(3) Å, c = 10.8192(4) Å, β = 93.4346(15)°, V = 664.15(4) Å³, Z = 2, Z' = 0.5, T = 100(2) K, d_{calc} = 1.357 g cm⁻³, μ(Mo *Kα*) = 0.084 mm⁻¹, F(000) = 284; $T_{max/min}$ = 0.9544/0.9140; 35628 reflections were collected (2.859° ≤ θ ≤ 30.755°, index ranges: –12 ≤ h ≤ 12, –9 ≤ k ≤ 9, and –15 ≤ l ≤ 15), 2228 of which were unique, R_{int} = 0.0484, $R_σ$ = 0.0230; completeness to θ of 30.755° 99.9 %. The refinement of 122 parameters with no restraints converged to $R1$ = 0.0419 and $wR2$ = 0.1147 for 1840 reflections with $I > 2σ(I)$ and $R1$ = 0.0527 and $wR2$ = 0.1228 for all data with goodness-of-fit S = 1.066 and residual electron density $ρ_{max/min}$ = 0.432 and –0.226 e Å⁻³, rms 0.056; max shift/e.s.d. in the last cycle 0.000.

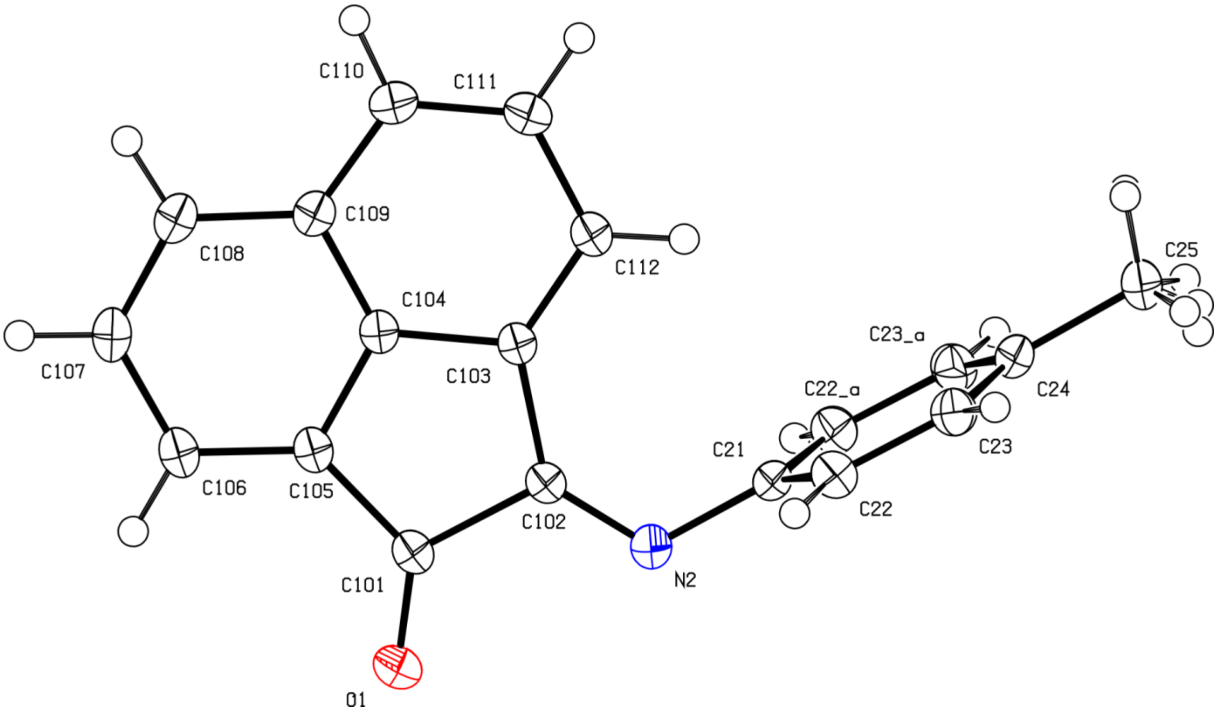

Crystallographic data for III.

$C_{19.5}H_{14}ClNO_2$, orange prism (0.366 × 0.306 × 0.098 mm³), formula weight 329.77 g mol⁻¹; triclinic, $P\overline{1}$ (No. 2), a = 6.8469(2) Å, b = 10.6889(4) Å, c = 11.7084(4) Å, α = 112.2666(8)°, β = 101.0078(8)°, γ = 92.0204(9)°, V = 772.92(5) Å³, Z = 2, Z' = 1, T = 119(2) K, d_{calc} = 1.417 g cm⁻³, μ(Mo $K\alpha$) = 0.258 mm⁻¹, F(000) = 342; $T_{max/min}$ = 0.9415/0.8808; 91866 reflections were collected (1.927° ≤ θ ≤ 33.769°, index ranges: −10 ≤ h ≤ 10, −16 ≤ k ≤ 16, and −18 ≤ l ≤ 18), 6191 of which were unique, R_{int} = 0.0419, R_{σ} = 0.0190; completeness to θ of 33.769° 99.8 %. The refinement of 227 parameters with 22 restraints converged to $R1$ = 0.0464 and $wR2$ = 0.1236 for 5082 reflections with $I > 2\sigma(I)$ and $R1$ = 0.0581 and $wR2$ = 0.1335 for all data with goodness-of-fit S = 1.028 and residual electron density $\varrho_{max/min}$ = 0.471 and −0.428 e Å⁻³, rms 0.054; max shift/e.s.d. in the last cycle 0.000.

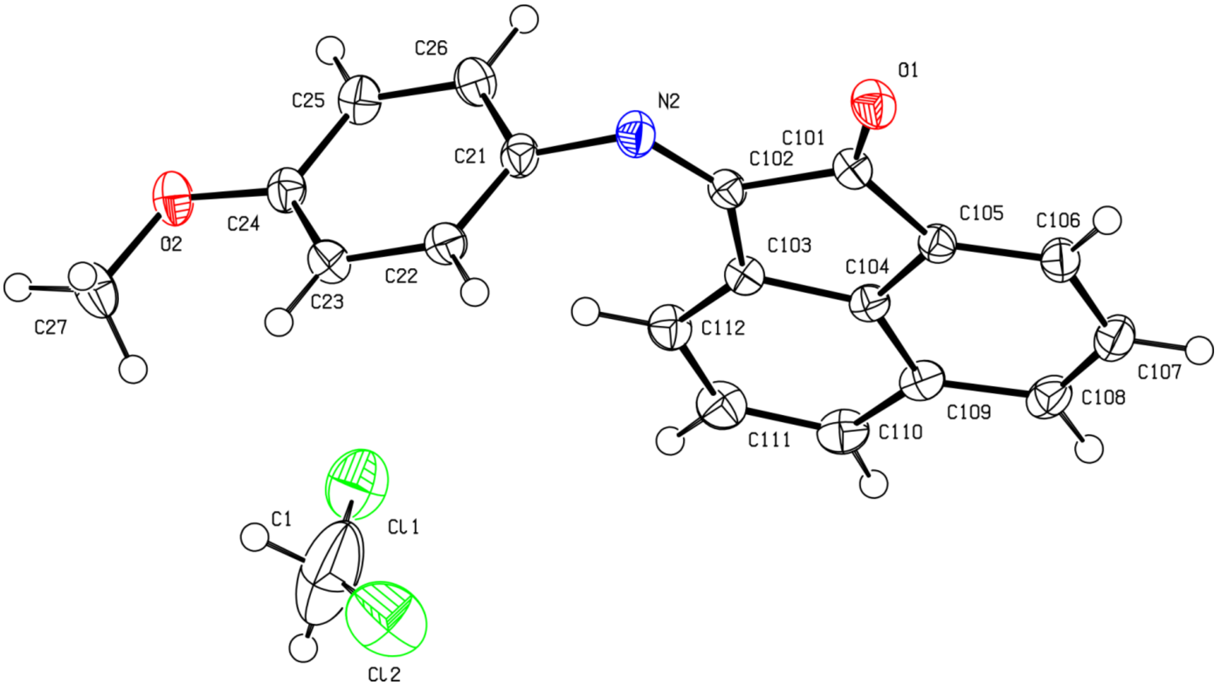

Crystallographic data for IX.

$C_{21}H_{12}N_2O$, orange prism (0.250 × 0.100 × 0.099 mm^3), formula weight 308.33 g mol^{-1}; monoclinic, $P2_1/c$ (No. 14), a = 8.0853(4) Å, b = 15.3987(8) Å, c = 11.7995(6) Å, β = 100.2989(19)°, V = 1445.41(13) Å^3, Z = 4, Z' = 1, T = 100(2) K, d_{calc} = 1.417 g cm^{-3}, μ(Mo $K\alpha$) = 0.089 mm^{-1}, F(000) = 640; $T_{max/min}$ = 0.9856/0.9516; 56865 reflections were collected (2.197° ≤ θ ≤ 30.977°, index ranges: –11 ≤ h ≤ 11, –21 ≤ k ≤ 22, and –16 ≤ l ≤ 16), 4563 of which were unique, R_{int} = 0.0730, R_σ = 0.0353; completeness to θ of 30.977° 99.2 %. The refinement of 217 parameters with no restraints converged to $R1$ = 0.0525 and $wR2$ = 0.1280 for 3507 reflections with $I > 2\sigma(I)$ and $R1$ = 0.0739 and $wR2$ = 0.1414 for all data with goodness-of-fit S = 1.050 and residual electron density $\varrho_{max/min}$ = 0.485 and –0.222 e Å^{-3}, rms 0.056; max shift/e.s.d. in the last cycle 0.000.

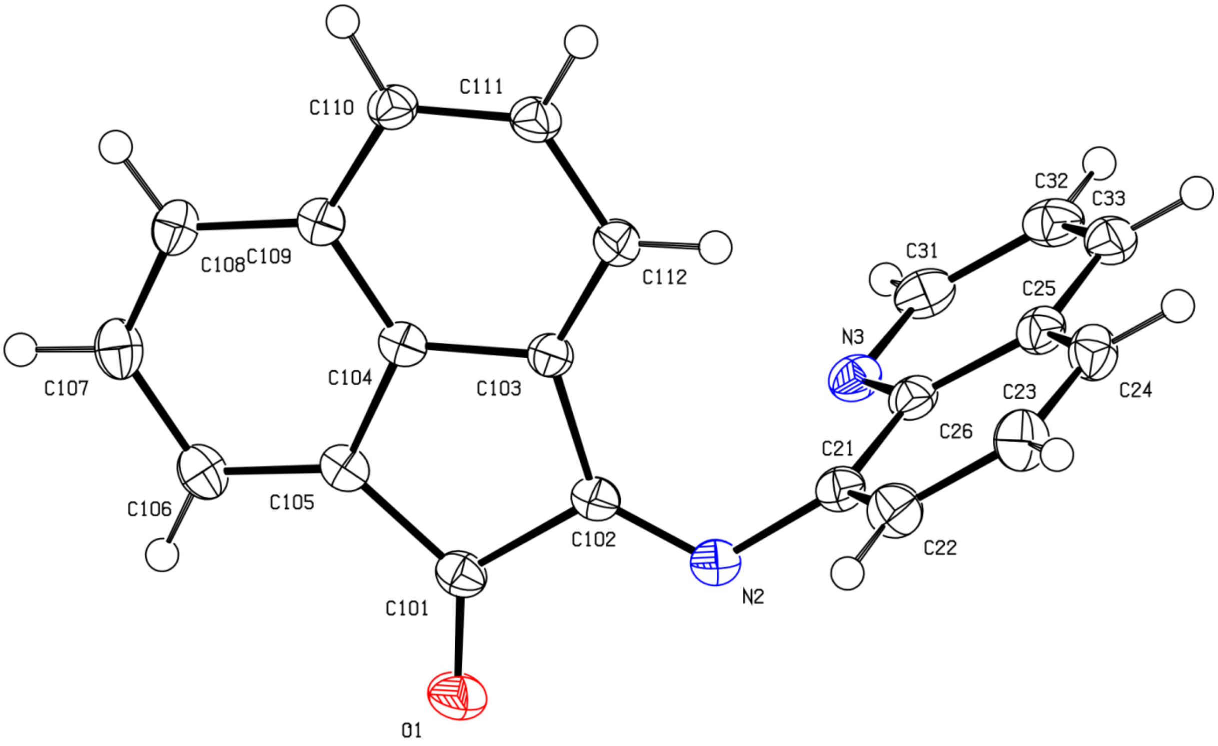

Crystallographic data for 1-NBu$_4$.

$C_{38}H_{56}N_2O_2$, prism (0.181 × 0.147 × 0.080 mm^3), formula weight 572.84 g mol^{-1}; monoclinic, $P2_1/n$ (No. 14), a = 13.3176(13) Å, b = 18.2881(16) Å, c = 15.4922(13) Å, β = 112.518(3)°, V = 3485.5(5) Å^3, Z = 4, Z' = 1, T = 150(2) K, d_{calc} = 1.092 g cm^{-3}, μ(Mo $K\alpha$) = 0.066 mm^{-1}, F(000) = 1256; $T_{max/min}$ = 1.0000/0.9488; 77179 reflections were collected (1.807° ≤ θ ≤ 25.348°, index ranges: –16 ≤ h ≤ 16, –22 ≤ k ≤ 22, and –18 ≤ l ≤ 18), 6382 of which were unique, R_{int} = 0.0622, R_σ = 0.0274; completeness to θ of 25.348° 100.0 %. The refinement of 434 parameters with 246 restraints converged to $R1$ = 0.0555 and $wR2$ = 0.1341 for 4805 reflections with $I > 2\sigma(I)$ and $R1$ = 0.0779 and $wR2$ = 0.1512 for all data with goodness-of-fit S = 1.038 and residual electron density $\varrho_{max/min}$ = 0.408 and –0.275 e Å^{-3}, rms 0.048; max shift/e.s.d. in the last cycle 0.000.

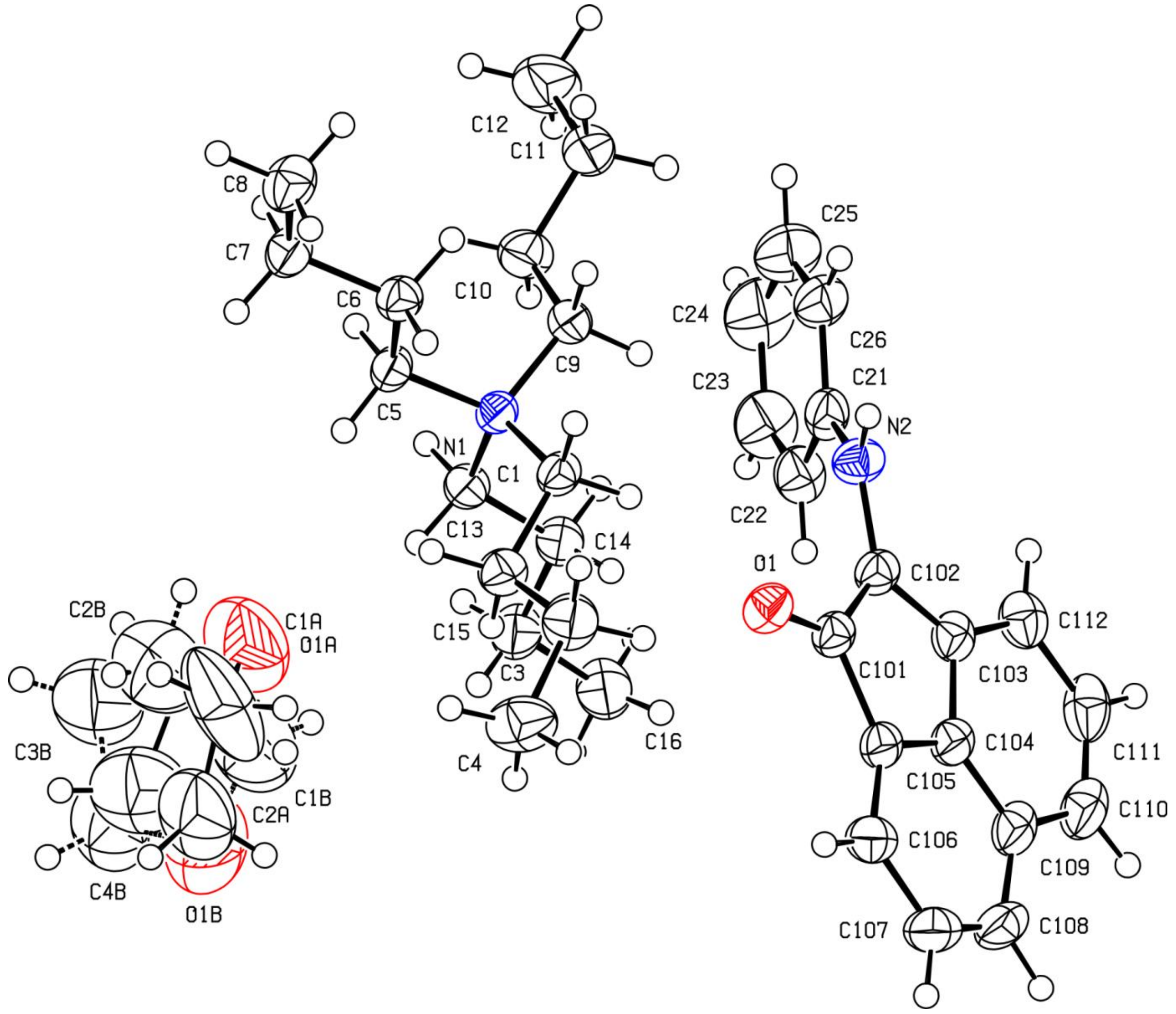

Crystallographic data for 3-NBu4.

$C_{37}H_{55}N_2O_{2.5}$, blue plate (0.16 × 0.07 × 0.05 mm^3), formula weight 567.83 g mol^{-1}; monoclinic, $P2_1/n$ (No. 14), a = 11.7563(9) Å, b = 19.2238(9) Å, c = 15.5867(10) Å, β = 106.480(8)°, V = 3377.9(4) $Å^3$, Z = 4, Z' = 1, T = 100.0(2) K, d_{calc} = 1.117 g cm^{-3}, μ(Cu $K\alpha$) = 0.529 mm^{-1}, F(000) = 1244; $T_{max/min}$ = 1.000/0.821; 22539 reflections were collected (3.746° ≤ θ ≤ 76.368°, index ranges: –14 ≤ h ≤ 14, –23 ≤ k ≤ 21, and –18 ≤ l ≤ 19), 6768 of which were unique, R_{int} = 0.0714, R_σ = 0.0664; completeness to θ of 76.368° 95.5 %. The refinement of 421 parameters with 4 restraints converged to $R1$ = 0.0565 and $wR2$ = 0.1416 for 4447 reflections with $I > 2\sigma(I)$ and $R1$ = 0.0899 and $wR2$ = 0.1581 for all data with goodness-of-fit S = 1.043 and residual electron density $\varrho_{max/min}$ = 0.271 and –0.205 e $Å^{-3}$, rms 0.042; max shift/e.s.d. in the last cycle 0.001.

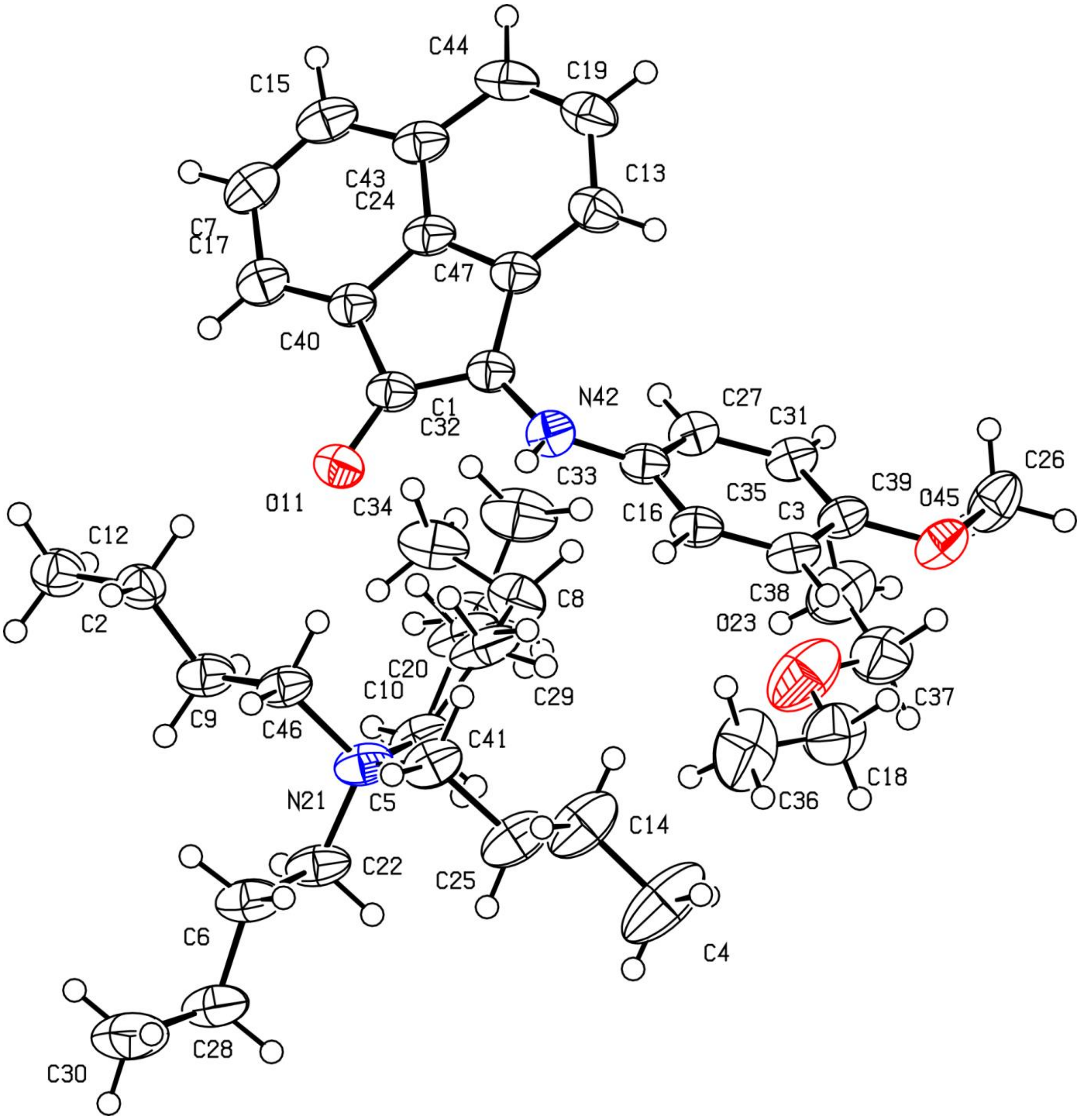

Crystallographic data for 5-Na.

$C_{104}H_{108}N_4Na_4O_8$, dark purple prism (0.331 × 0.238 × 0.08 mm³), formula weight 1633.90 g mol⁻¹; triclinic, $P\overline{1}$ (No. 2), a = 20.2236(3) Å, b = 22.0193(4) Å, c = 25.4074(4) Å, α = 71.8195(3)°, β = 70.3564(3)°, γ = 85.0224(3)°, V = 10121.7(3) Å³, Z = 4, Z' = 2, T = 150.00 K, d_{calc} = 1.072 g cm⁻³, μ(Mo $K\alpha$) = 0.082 mm⁻¹, $F(000)$ = 3472; $T_{max/min}$ = 0.9521/0.9147; 566343 reflections were collected (1.431° ≤ θ ≤ 25.398°, index ranges: –24 ≤ h ≤ 24, –26 ≤ k ≤ 26, and –30 ≤ l ≤ 30), 37222 of which were unique, R_{int} = 0.0549, R_{σ} = 0.0262; completeness to θ of 25.398° 99.8 %. The refinement of 2292 parameters with 649 restraints converged to $R1$ = 0.0617 and $wR2$ = 0.1883 for 27477 reflections with $I > 2\sigma(I)$ and $R1$ = 0.0795 and $wR2$ = 0.2077 for all data with goodness-of-fit S = 1.052 and residual electron density $\rho_{max/min}$ = 0.773 and –0.458 e Å⁻³, rms 0.054; max shift/e.s.d. in the last cycle 0.001.

Crystallographic data for 6-Na.

$C_{120}H_{148}N_4Na_4O_{10}$, black cube (0.26 × 0.25 × 0.23 mm³), formula weight 1898.38 g mol⁻¹; cubic, $Ia\overline{3}$ (No. 206), a = 41.6384(3) Å, b = 41.6384(3) Å, c = 41.6384(3) Å, V = 72190.9(14) Å³, Z = 24, Z' = 0.5, T = 99.98(11) K, d_{calc} = 1.048 g cm⁻³, μ(Cu $K\alpha$) = 0.638 mm⁻¹, $F(000)$ = 24480; $T_{max/min}$ = 1.000/0.881; 43289 reflections were collected (2.599° ≤ θ ≤ 76.148°, index ranges: –45 ≤ h ≤ 51, –19 ≤ k ≤ 40, and –12 ≤ l ≤ 50), 12071 of which were unique, R_{int} = 0.0456, R_{σ} = 0.0430; completeness to θ of 76.148° 95.7 %. The

refinement of 653 parameters with 65 restraints converged to $R1 = 0.0662$ and $wR2 = 0.2012$ for 9223 reflections with $I > 2\sigma(I)$ and $R1 = 0.0818$ and $wR2 = 0.2165$ for all data with goodness-of-fit $S = 1.054$ and residual electron density $\varrho_{max/min} = 0.719$ and −0.575 e Å^{-3}, rms 0.056; max shift/e.s.d. in the last cycle 0.001.

Crystallographic data for 4-NBu₄.

$C_{56}H_{69}N_3O_2$, purple block (0.49 × 0.29 × 0.2 mm^3), formula weight 816.14 g mol^{-1}; triclinic, $P\overline{1}$ (No. 2), $a = 8.74946(12)$ Å, $b = 12.76812(18)$ Å, $c = 22.9415(3)$ Å, $\alpha = 75.1593(12)°$, $\beta = 82.8769(11)°$, $\gamma = 71.7374(13)°$, $V = 2349.94(6)$ Å^3, $Z = 2$, $Z' = 1$, $T = 100.0(2)$ K, $d_{calc} = 1.153$ g cm^{-3}, $\mu(\text{Cu } K\alpha) = 0.528$ mm^{-1}, $F(000) = 884$; $T_{max/min} = 1.000/0.331$; 28567 reflections were collected ($1.994° \leq \theta \leq 76.569°$, index ranges: $-9 \leq h \leq 11$, $-16 \leq k \leq 16$, and $-28 \leq l \leq 26$), 9544 of which were unique, $R_{int} = 0.0461$, $R_\sigma = 0.0541$; completeness to θ of 76.569° 96.7 %. The refinement of 568 parameters with no restraints converged to $R1 = 0.0443$ and $wR2 = 0.1200$ for 8214 reflections with $I > 2\sigma(I)$ and $R1 = 0.0537$ and $wR2 = 0.1255$ for all data with goodness-of-fit $S = 1.031$ and residual electron density $\varrho_{max/min} = 0.248$ and −0.208 e Å^{-3}, rms 0.042; max shift/e.s.d. in the last cycle 0.001.

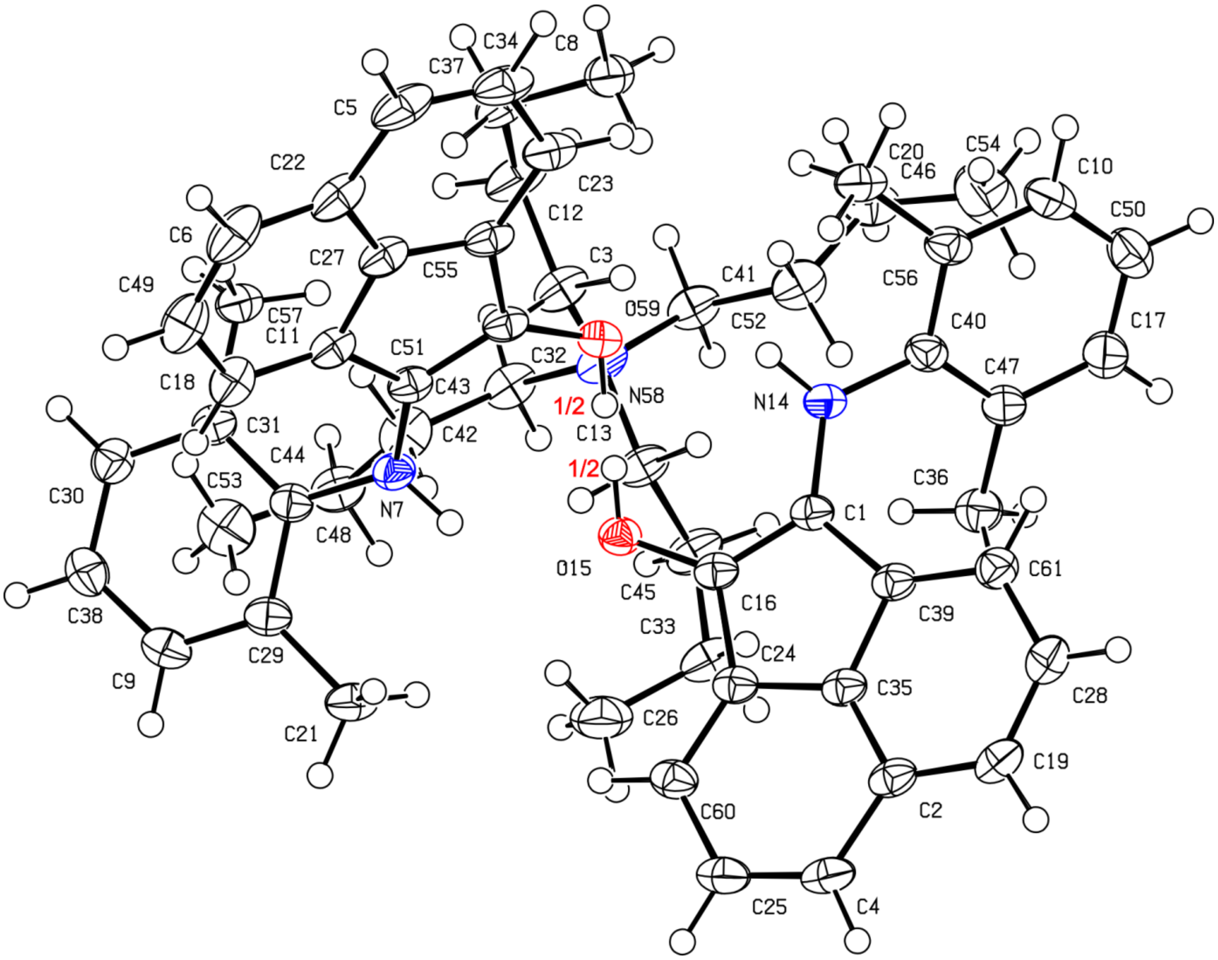

Crystallographic data for 7-Na.

$C_{100}H_{100}N_4Na_4O_8$, black cube (0.29 × 0.28 × 0.22 mm^3), formula weight 1577.79 g mol^{-1}; tetragonal, $P\overline{4}2_1c$ (No. 114), a = 18.8370(3) Å, $b = 18.8370(3)$ Å, $c = 29.6445(5)$ Å, $V = 10518.9(3)$ Å^3, $Z = 4$, $Z' = 2$, $T = 100.0(2)$ K, $d_{calc} = 0.996$ g cm^{-3}, $\mu(\text{Cu } K\alpha) = 0.637$ mm^{-1}, $F(000) = 3344$; $T_{max/min} = 1.000/0.255$; 33386 reflections were collected ($2.779° \leq \theta \leq 75.919°$, index ranges: $-23 \leq h \leq 22$, $-18 \leq k \leq 23$, and $-36 \leq l \leq 14$), 10223 of which were unique, $R_{int} = 0.0450$, $R_\sigma = 0.0378$; completeness to θ of 75.919° 98.0 %. The refinement of 536 parameters with 196 restraints converged to $R1 = 0.1142$ and $wR2 = 0.2919$ for 7688 reflections with $I > 2\sigma(I)$ and $R1 = 0.1313$ and $wR2 = 0.3153$ for all data with goodness-of-fit $S = 1.243$ and residual electron density $\varrho_{max/min} = 0.867$ and −0.443 e Å^{-3}, rms 0.116; max shift/e.s.d. in the last cycle 0.000.

Crystallographic data for 5-NaBH$_4$.

$C_{104}H_{112}N_4Na_4O_8$, orange plate (0.126 × 0.114 × 0.017 mm^3), formula weight 1637.93 g mol^{-1}; monoclinic, *P*2/*n* (No. 13), a = 25.7746(9) Å, b = 13.4370(5) Å, c = 26.7280(9) Å, β = 108.5738(12)°, V = 8774.6(5) Å^3, Z = 4, Z' = ½ + ½, T = 100(2) K, d_{calc} = 1.240 g cm^{-3}, μ(Mo $K\alpha$) = 0.094 mm^{-1}, F(000) = 3488; $T_{max/min}$ = 0.9862/0.9147; 131538 reflections were collected (1.329° ≤ θ ≤ 25.374°, index ranges: –31 ≤ h ≤ 30, –16 ≤ k ≤ 16, and –32 ≤ l ≤ 32), 16091 of which were unique, R_{int} = 0.1284, R_σ = 0.0807; completeness to θ of 25.374° 99.9 %. The refinement of 1105 parameters with 4 restraints converged to $R1$ = 0.0634 and $wR2$ = 0.1479 for 9558 reflections with $I > 2\sigma(I)$ and $R1$ = 0.1188 and $wR2$ = 0.1776 for all data with goodness-of-fit S = 1.040 and residual electron density $\rho_{max/min}$ = 0.646 and –0.426 e Å^{-3}, rms 0.065; max shift/e.s.d. in the last cycle 0.000.

REFERENCES

[1] Sheldrick, G. M. *SHELXT* – Integrated Space-Group and Crystal-Structure Determination. *Acta Crystallogr., Sect. A: Found. Adv.* **2015**, 71, 3–8.

[2] Sheldrick, G. M. Crystal Structure Refinement with *SHELXL*. *Acta Crystallogr., Sect. C: Struct. Chem.* **2015**, 71, 3–8.

[3] Farrugia, L. J. *WinGX* and *ORTEP* for Windows: An Update. *J. Appl. Crystallogr.* **2012**, 45, 849–854.

[4] Dolomanov, O. V.; Bourhis, L. J.; Gildea, R. J, Howard, J. A. K.; Puschmann, H. *OLEX2*: A Complete Structure Solution, Refinement and Analysis Program. *J. Appl. Crystallogr.* **2009**, 42, 339–341.

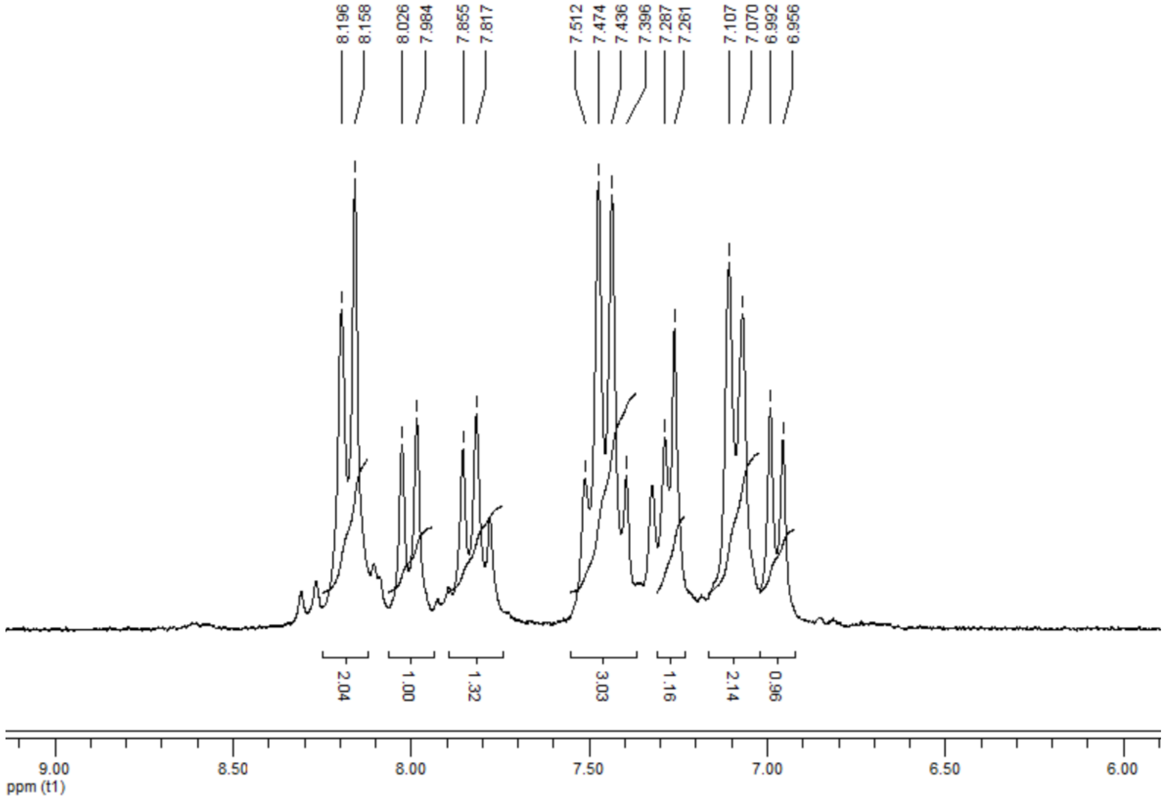

Figure S1. ^{1}H NMR spectrum of **I** in $CDCl_3$ (200 mHz).

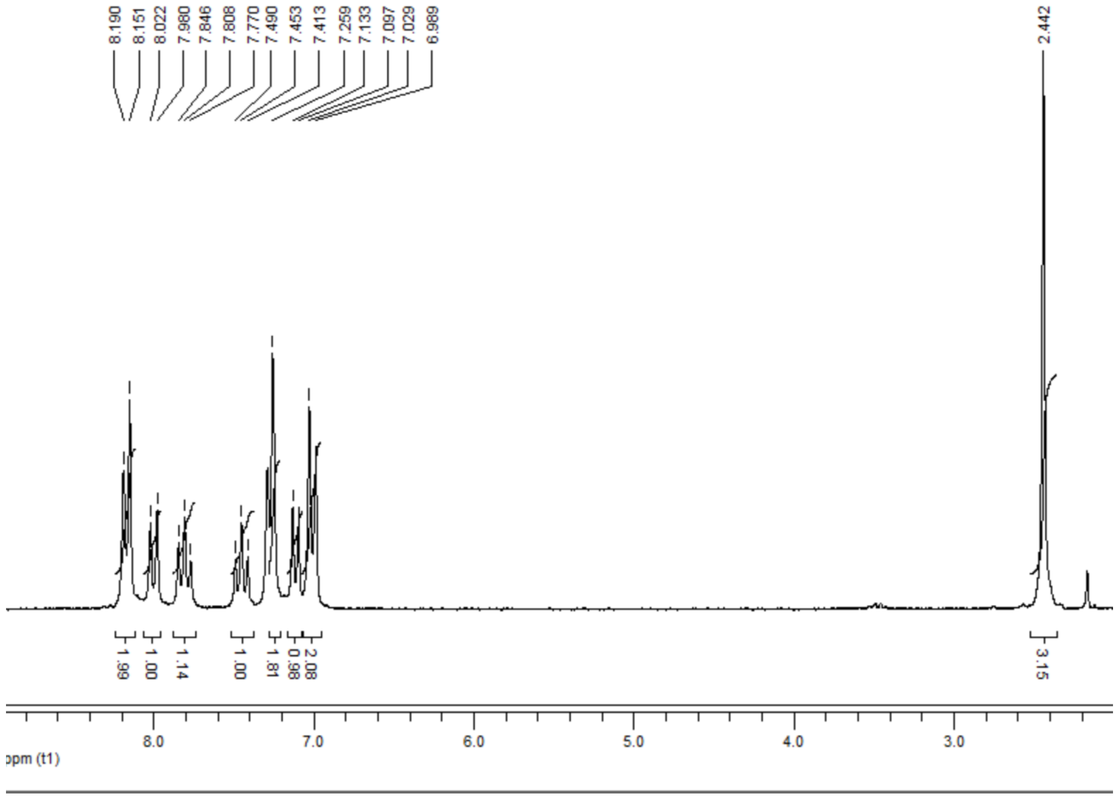

Figure S2. ^{1}H NMR spectrum of **II** in $CDCl_3$ (200 mHz).

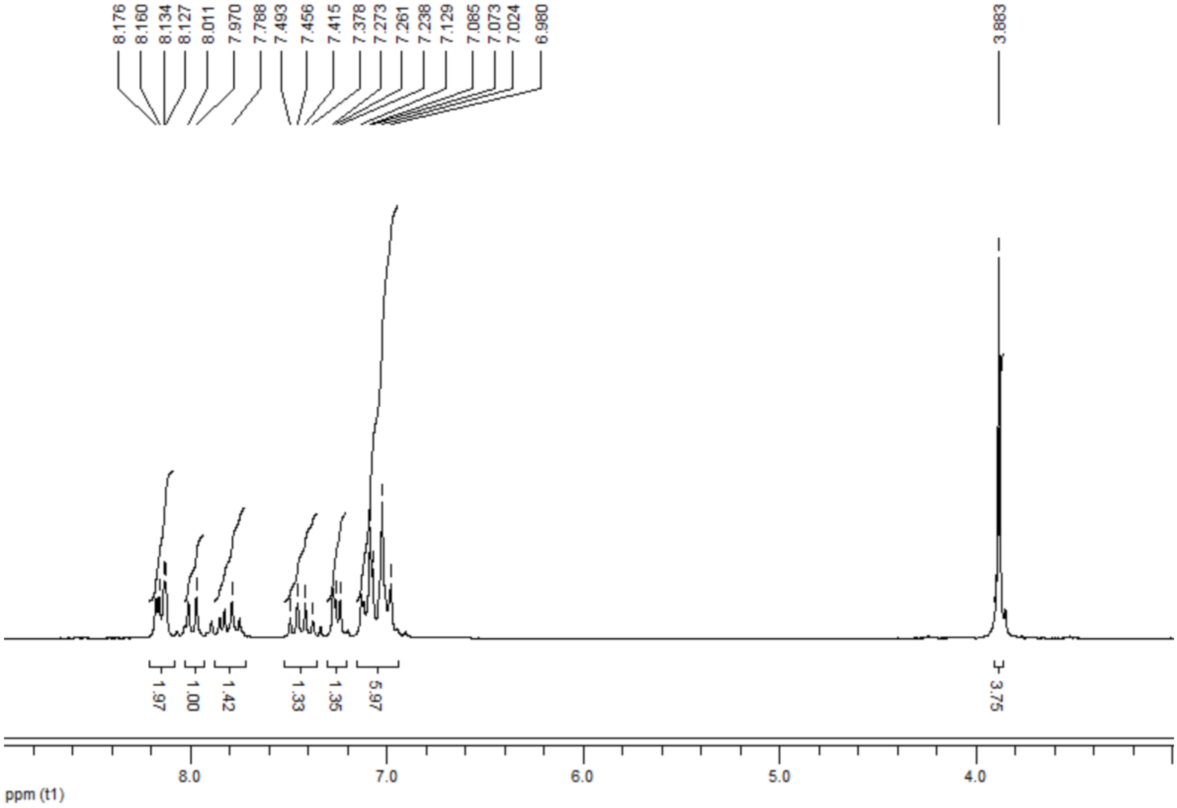

Figure S3. ^{1}H NMR spectrum of **III** in $CDCl_3$ (200 mHz).

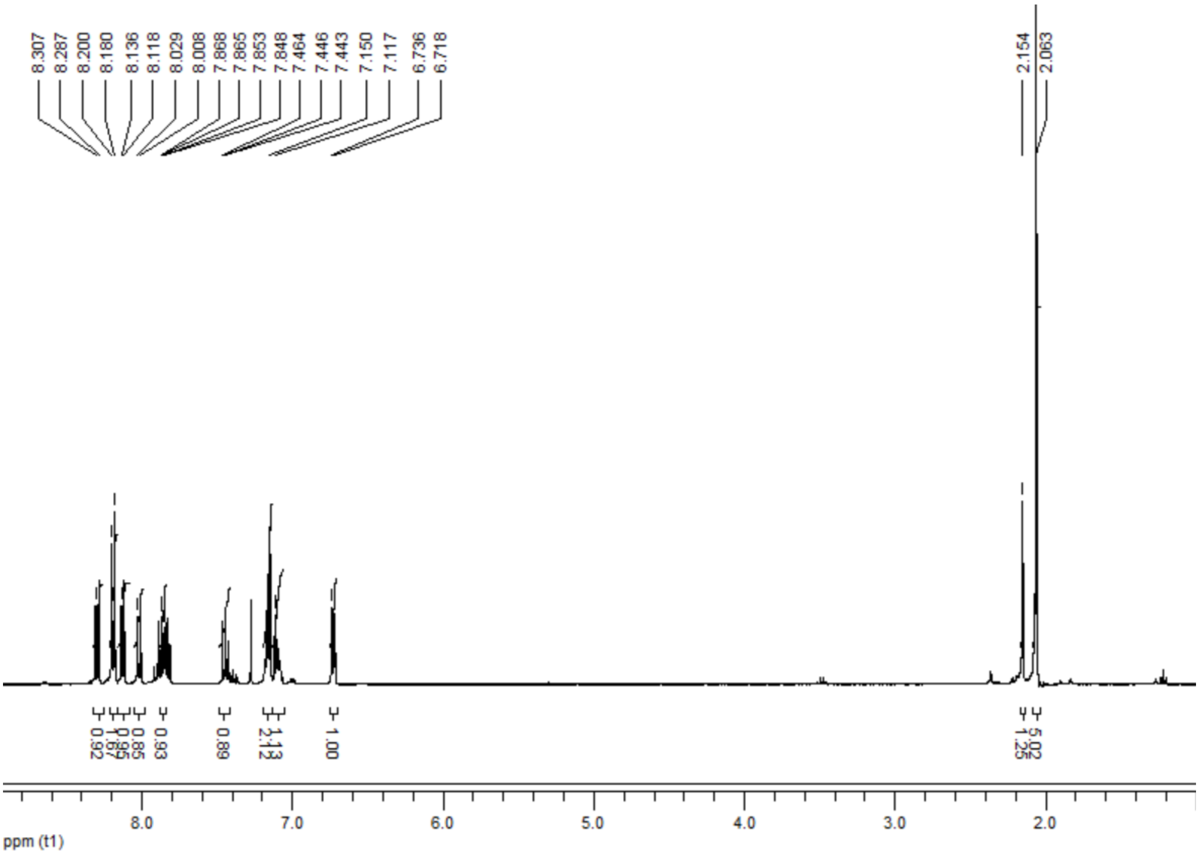

Figure S4. ^{1}H NMR spectrum of **IV** in $CDCl_3$ (400 mHz).

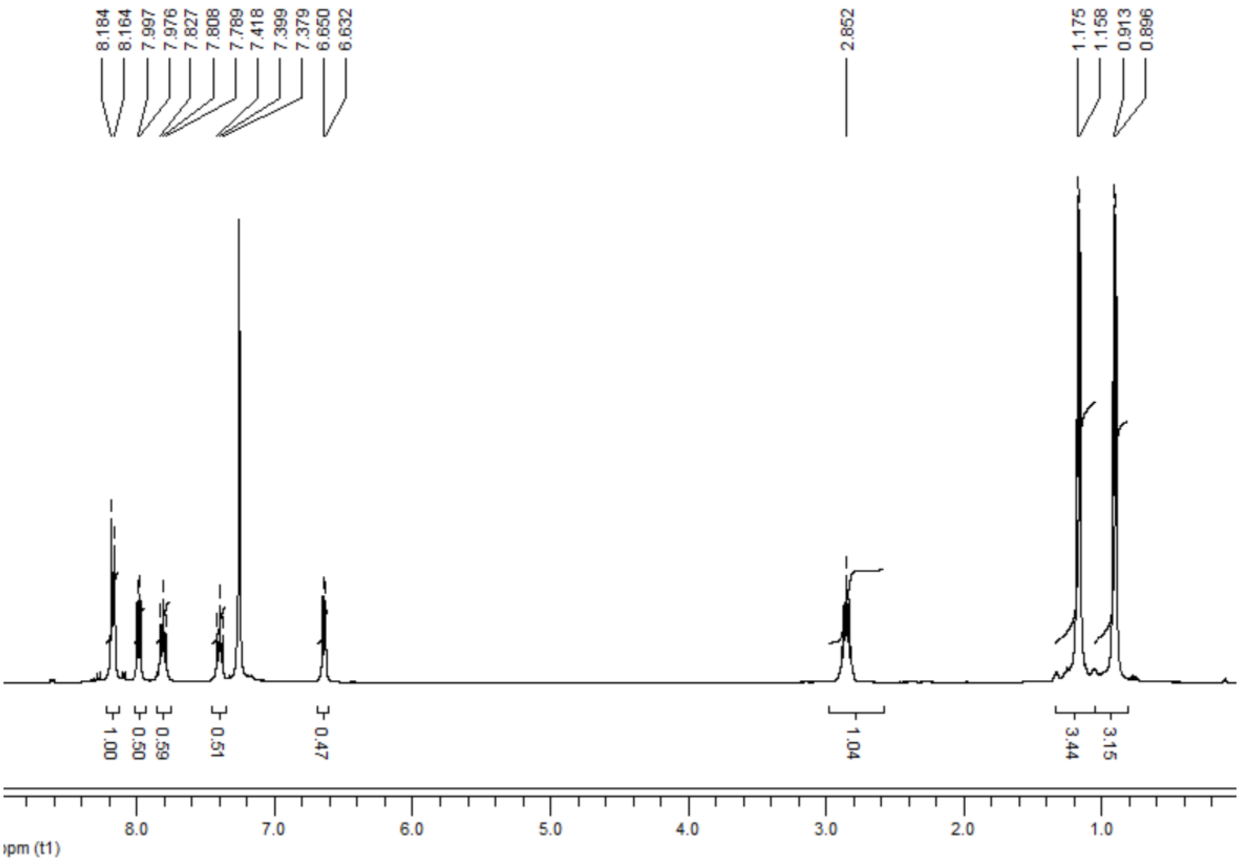

Figure S5. ^{1}H NMR spectrum of **V** in $CDCl_3$ (400 mHz).

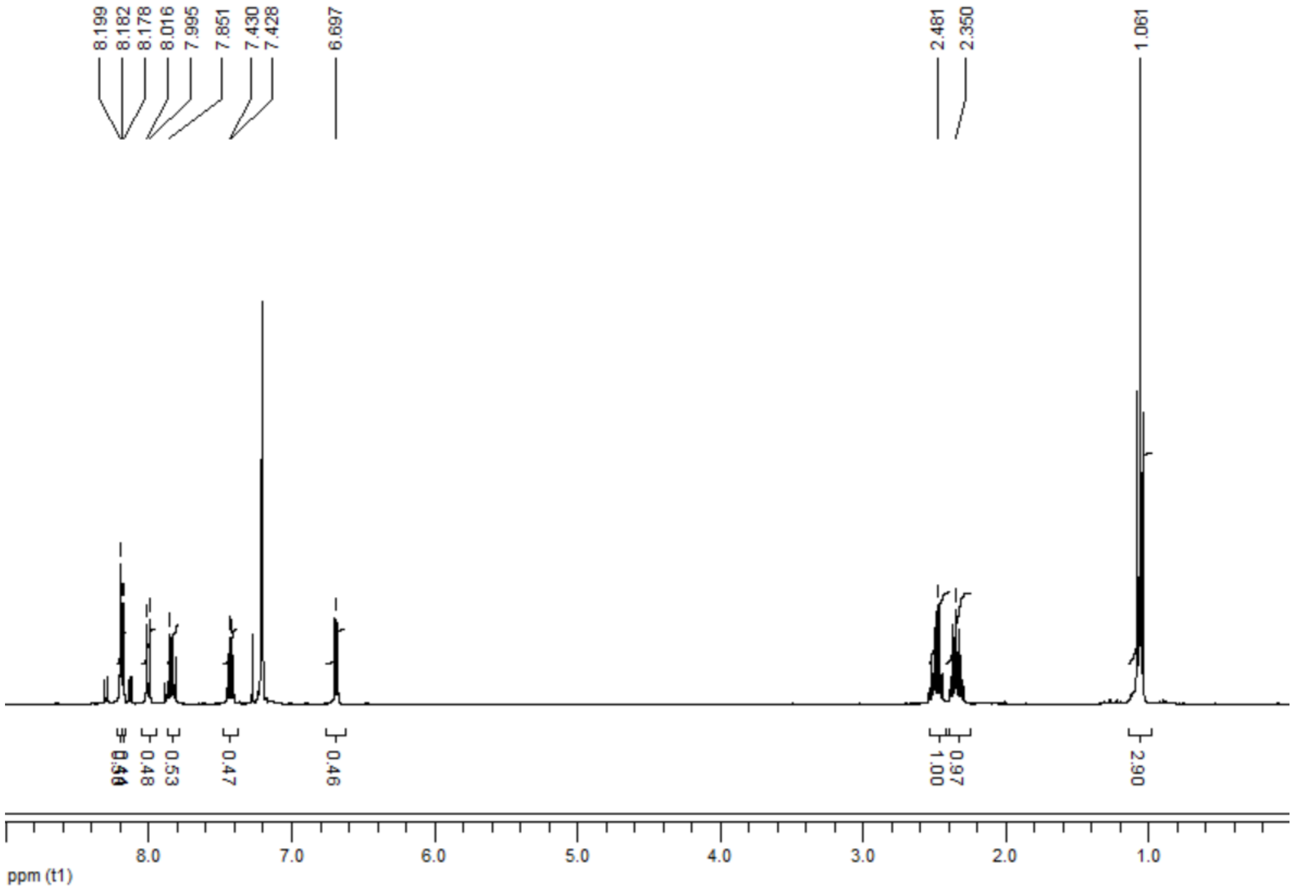

Figure S6. ^{1}H NMR spectrum of **VI** in $CDCl_3$ (400 mHz).

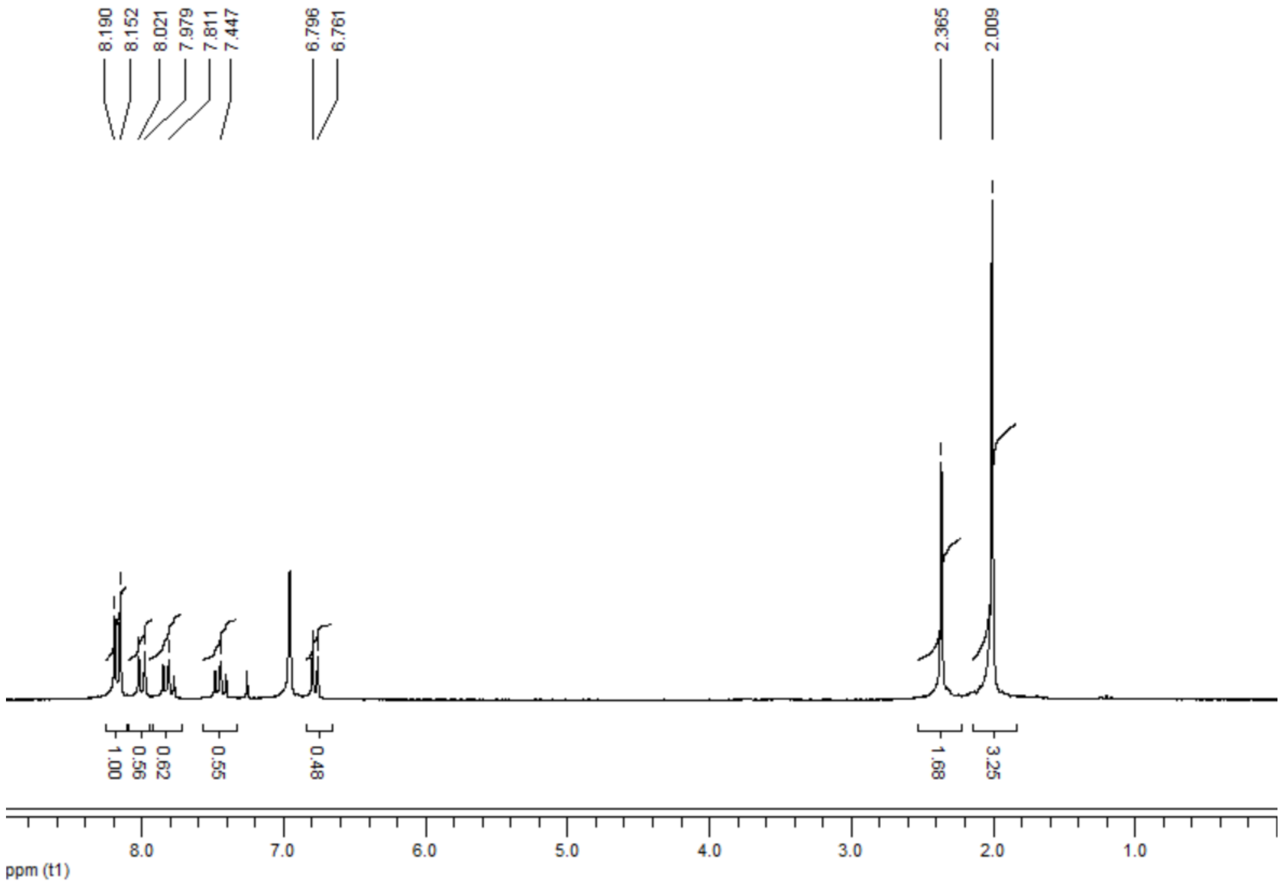

Figure S7. ^{1}H NMR spectrum of **VI** in $CDCl_3$ (400 mHz).

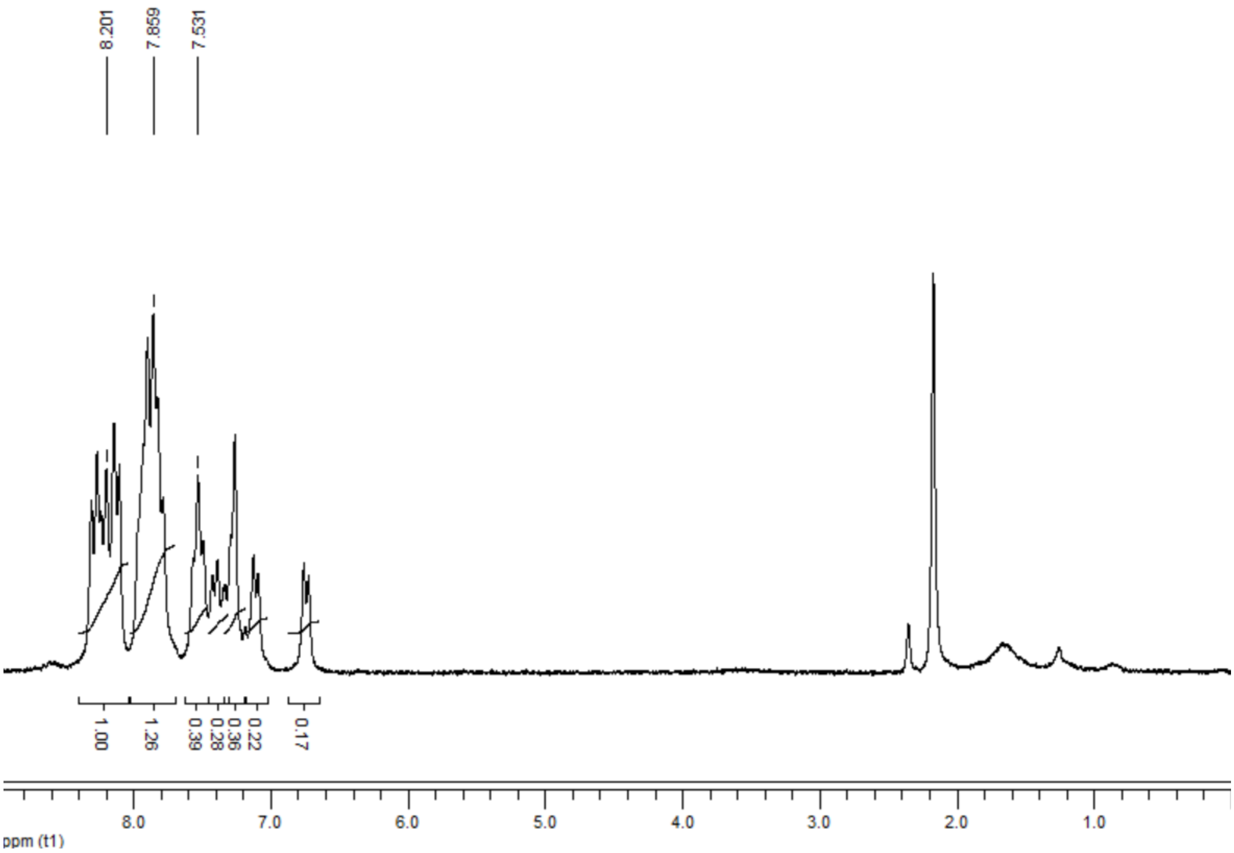

Figure S8. ^{1}H NMR spectrum of **VIII** in $CDCl_3$ (200 mHz).

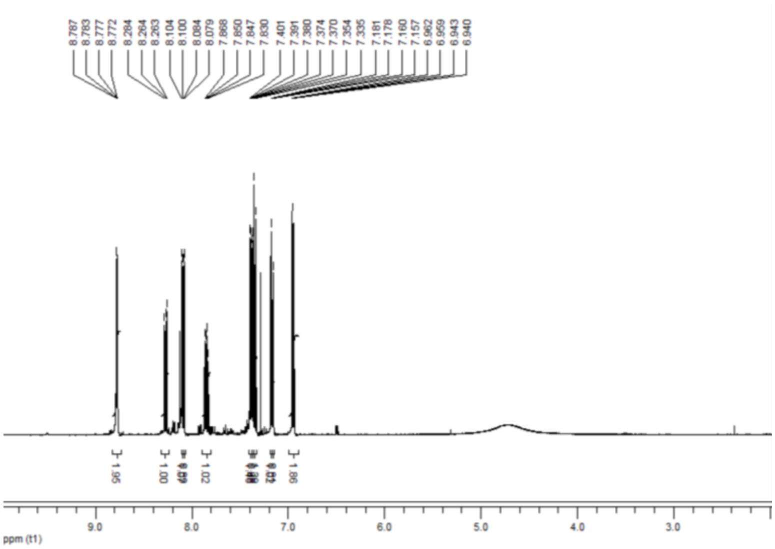

Figure S9. ^{1}H NMR spectrum of **IX** in $CDCl_3$ (400 mHz).

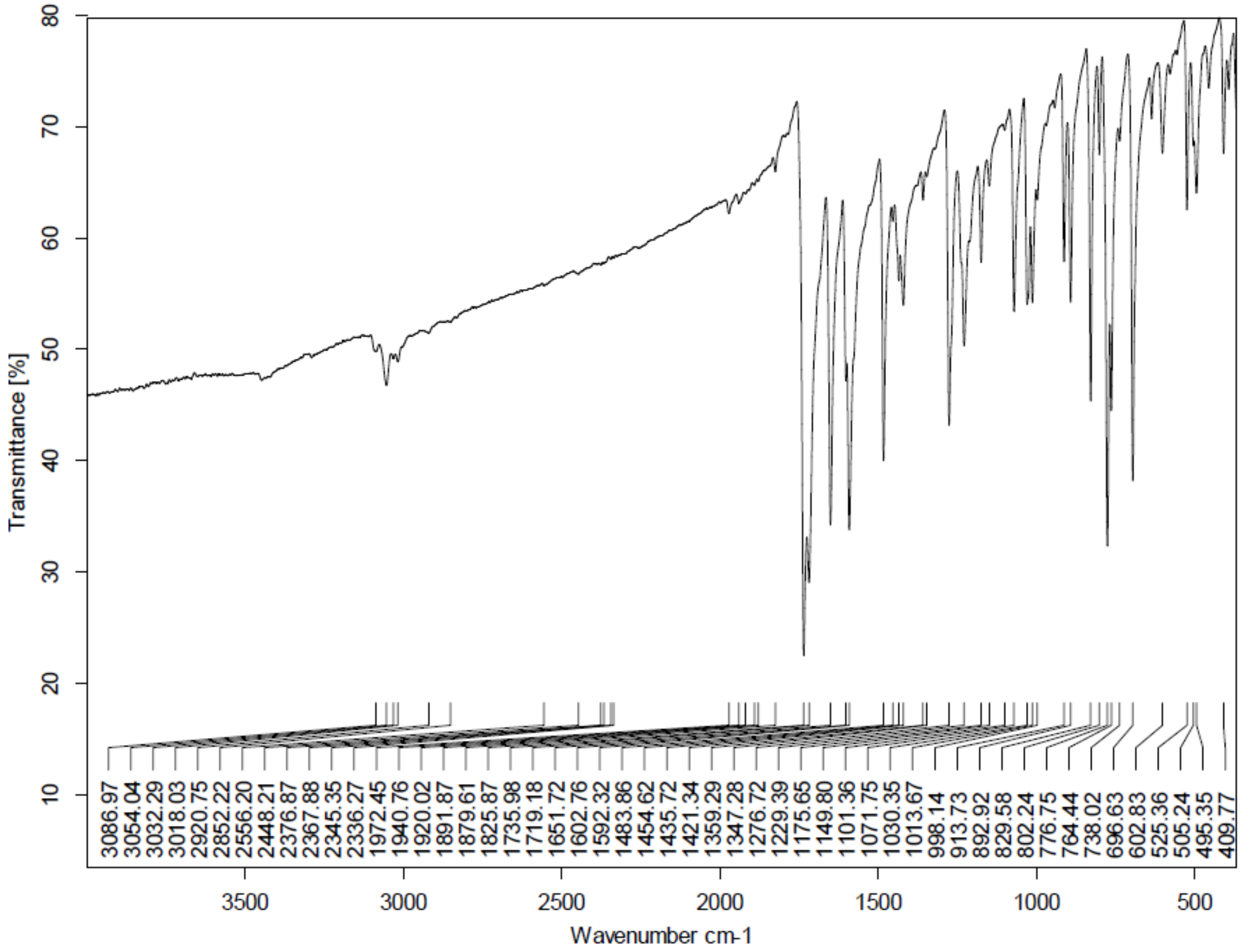

Figure S10. IR spectra of compound **I** in KBr.

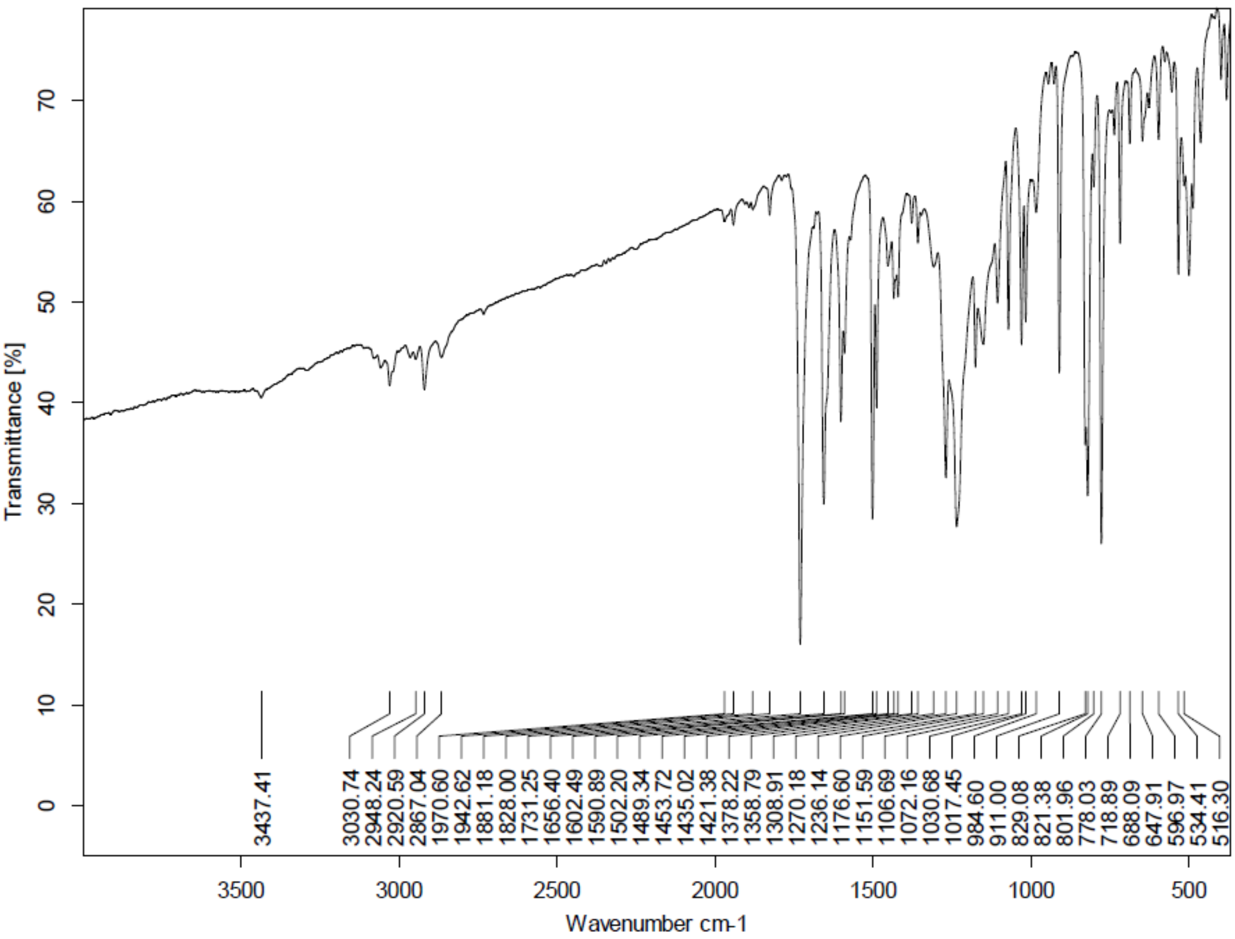

Figure S11. IR spectra of compound **II** in KBr.

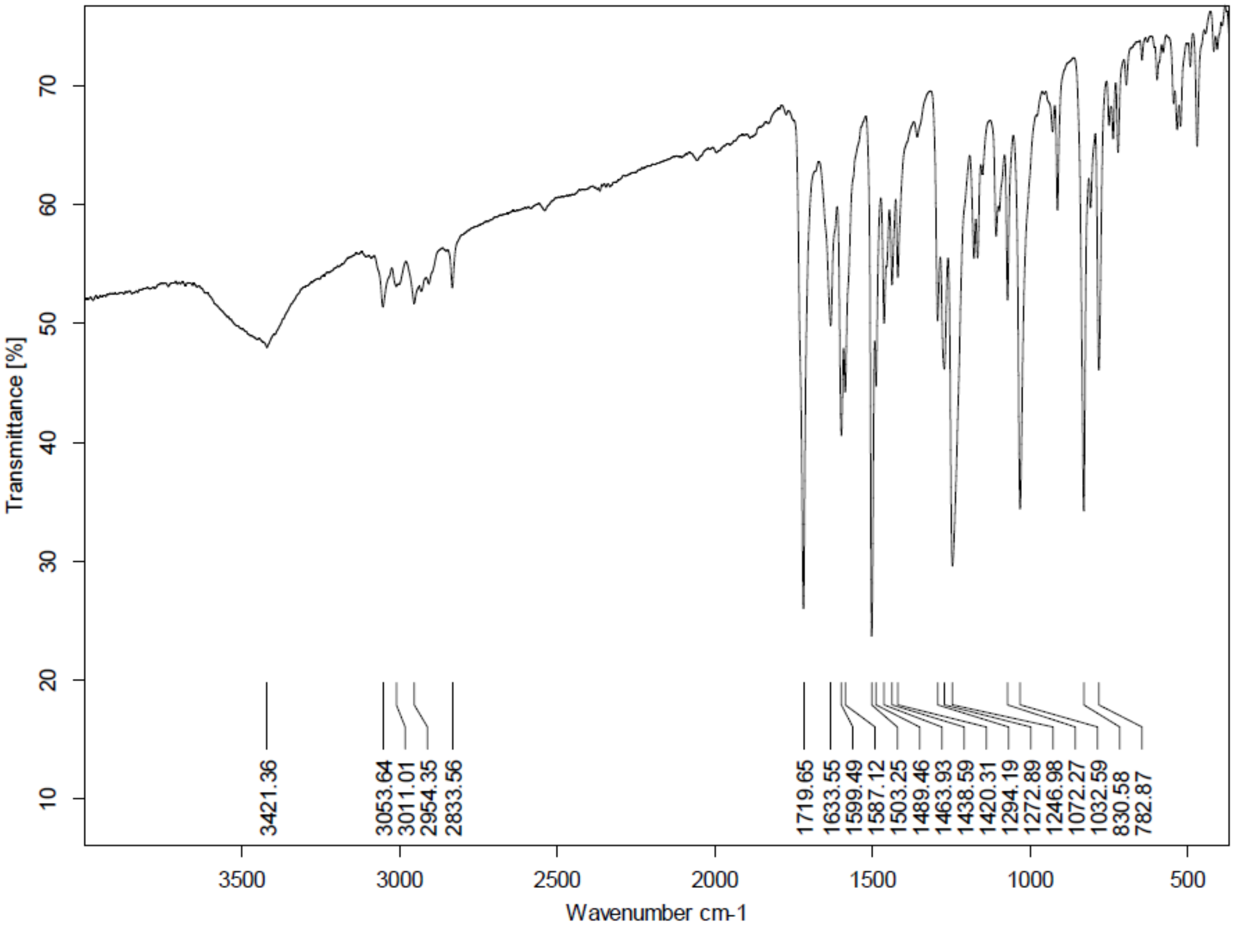

Figure S12. IR spectra of compound **III** in KBr.

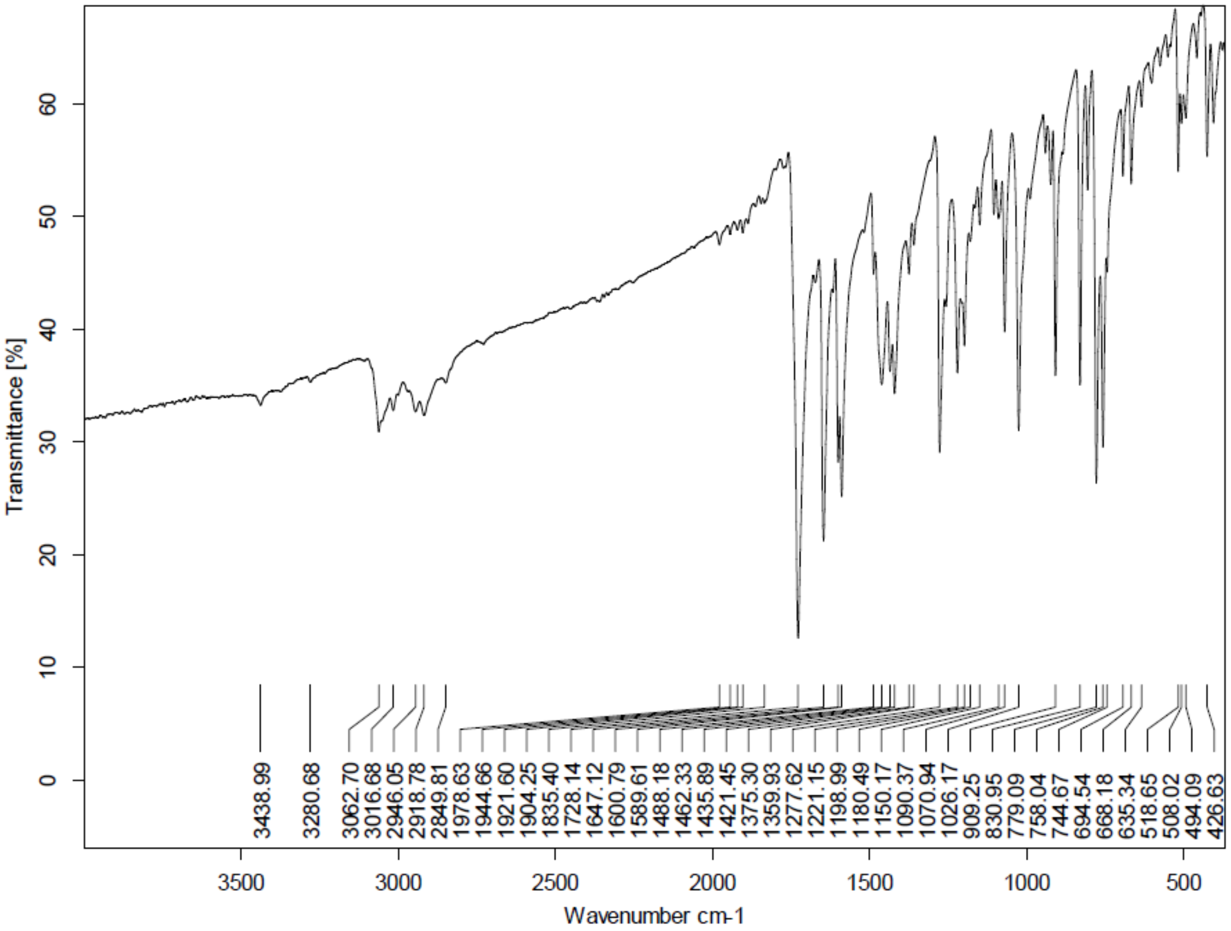

Figure S13. IR spectra of compound **IV** in KBr.

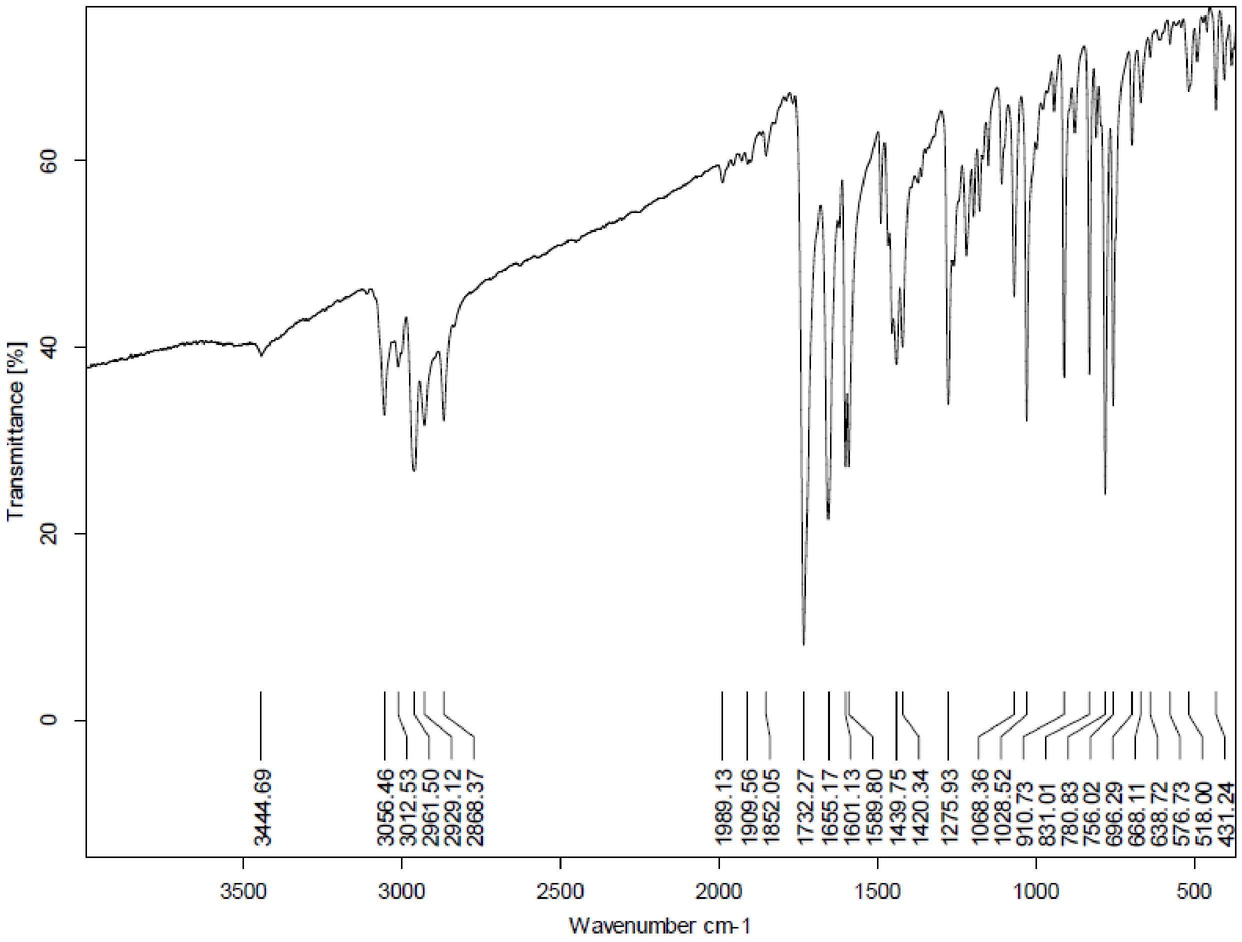

Figure S14. IR spectra of compound **V** in KBr.

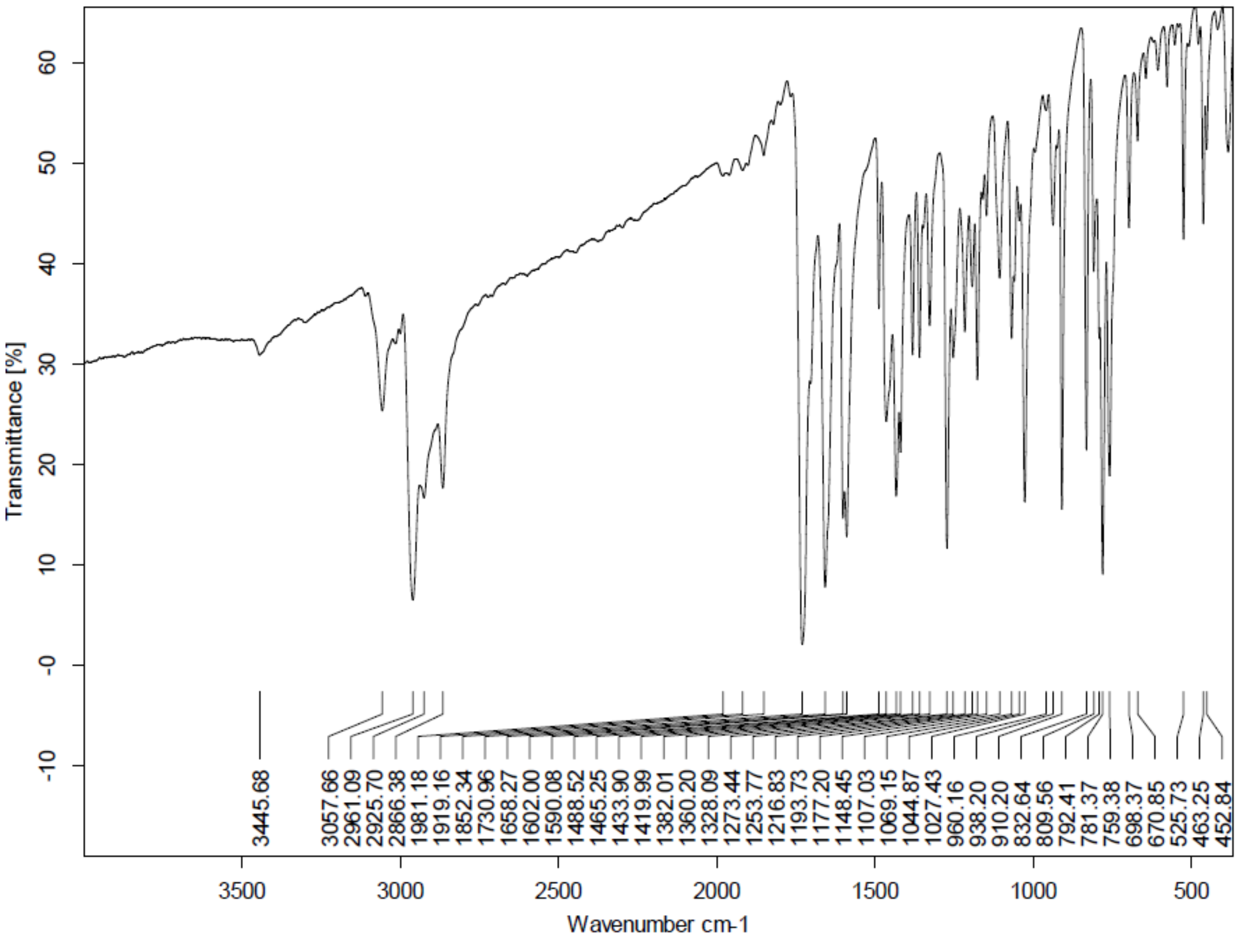

Figure S15. IR spectra of compound **VI** in KBr.

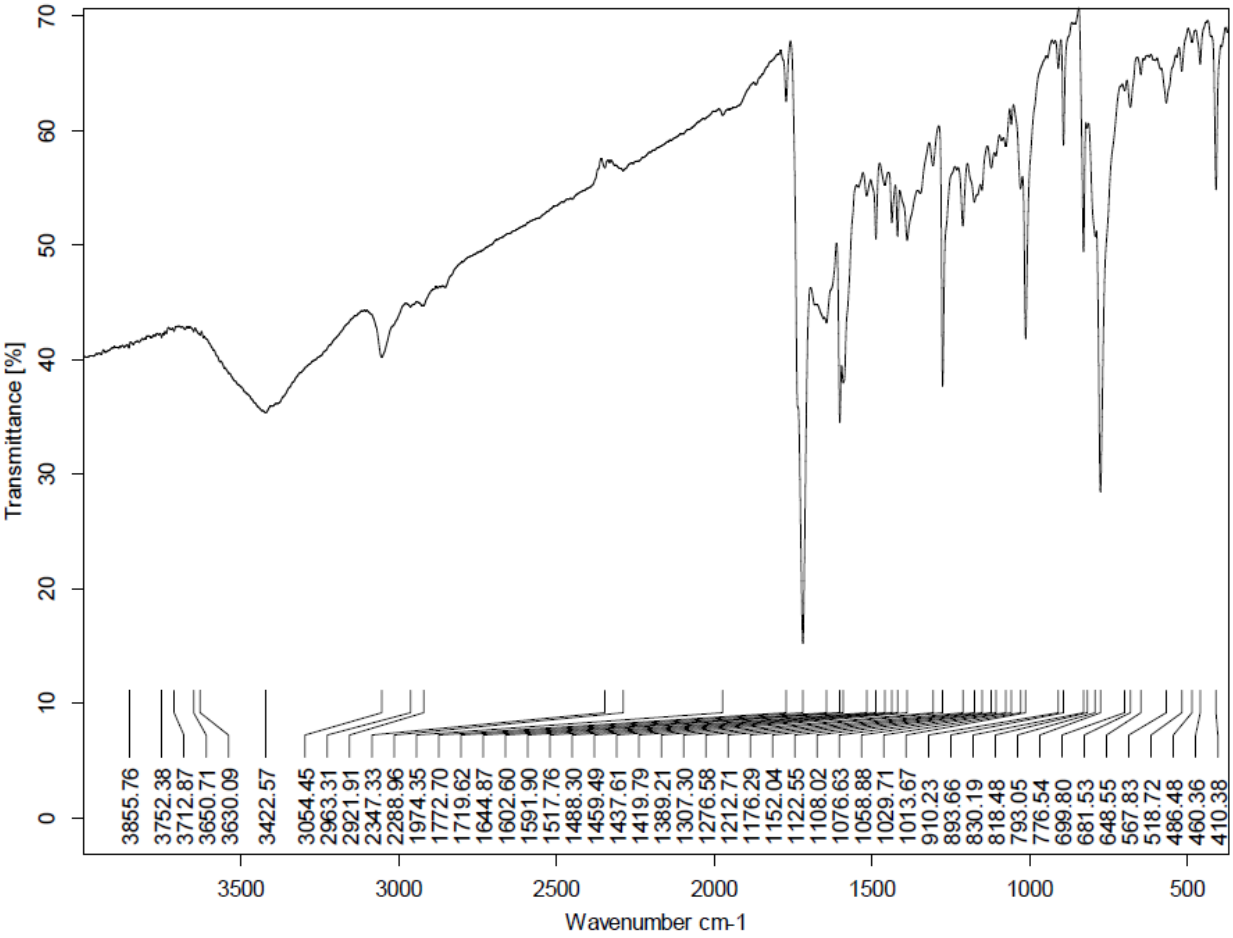

Figure S16. IR spectra of compound **VII** in KBr.

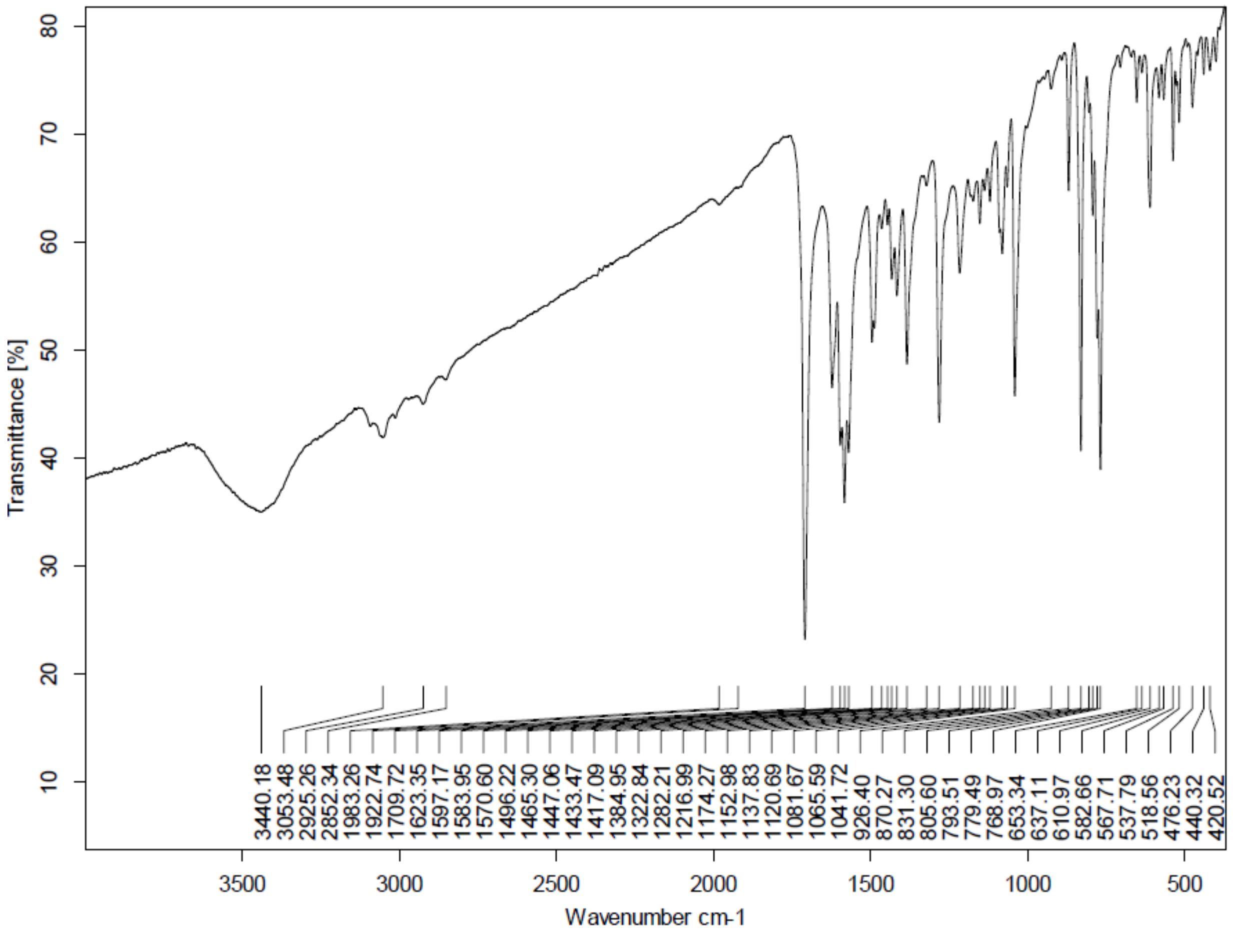

Figure S17. IR spectra of compound **VIII** in KBr.

Table S1. Frontiers orbitals for neutral MIANs I-IX.

	HOMO	LUMO
**I**	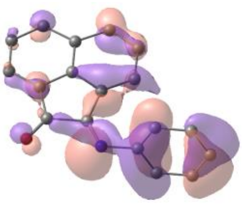	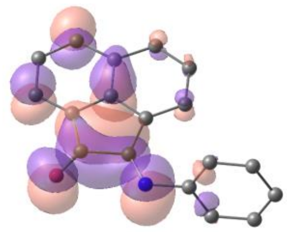
**III**	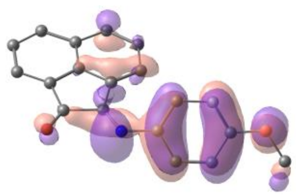	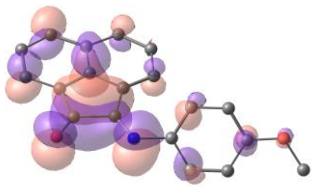

**IV**	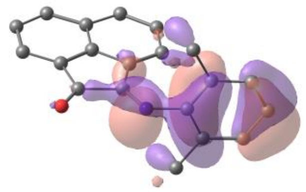	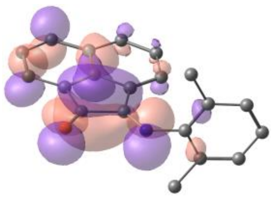
**V**	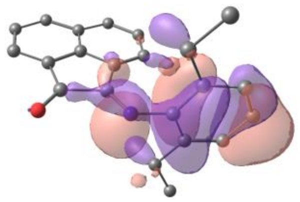	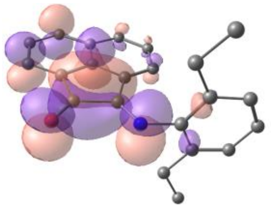
**VI**	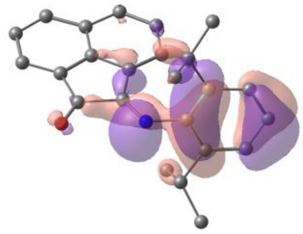	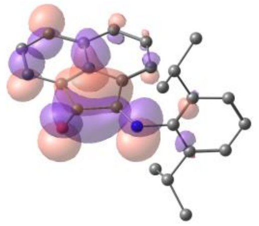
**VII**	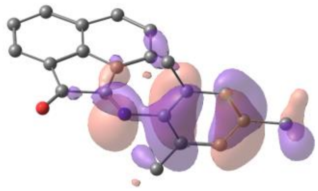	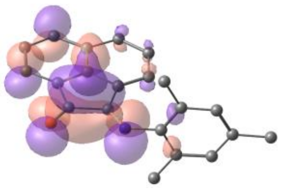
**VIII**	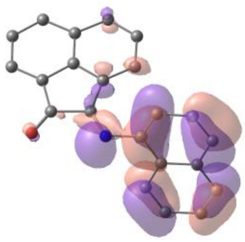	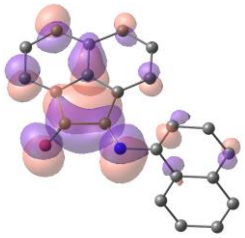
**IX**	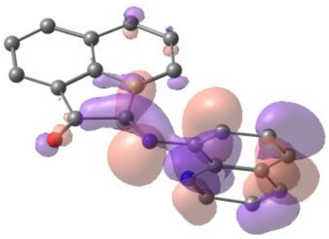	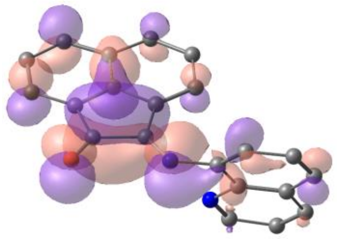

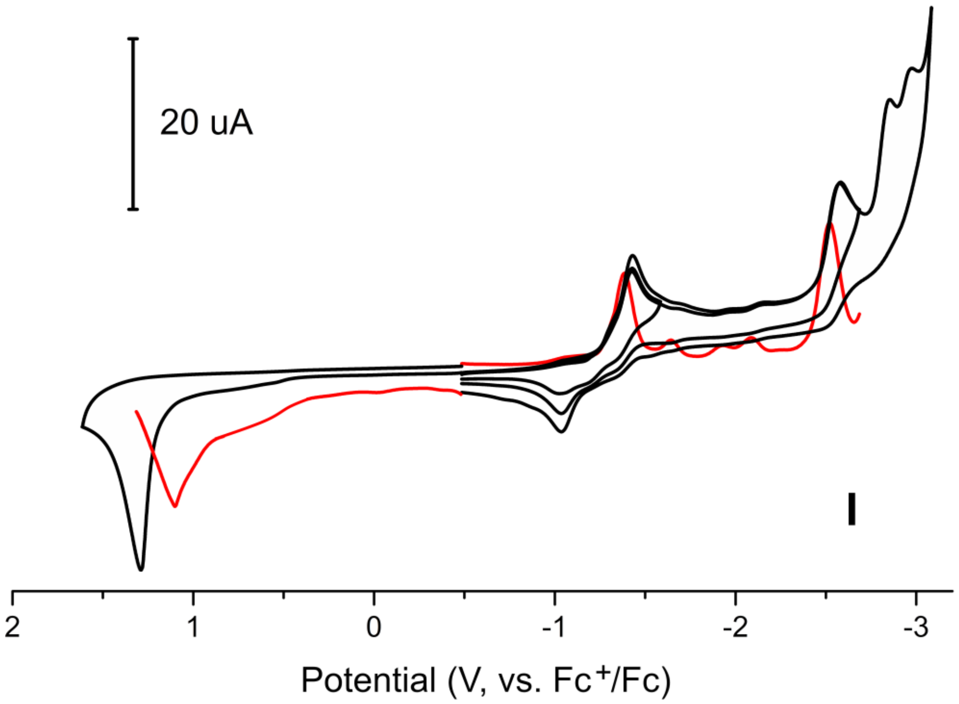
20 uA
I
2
1
0
-1
-2
-3
Potential (V, vs. Fc+/Fc)

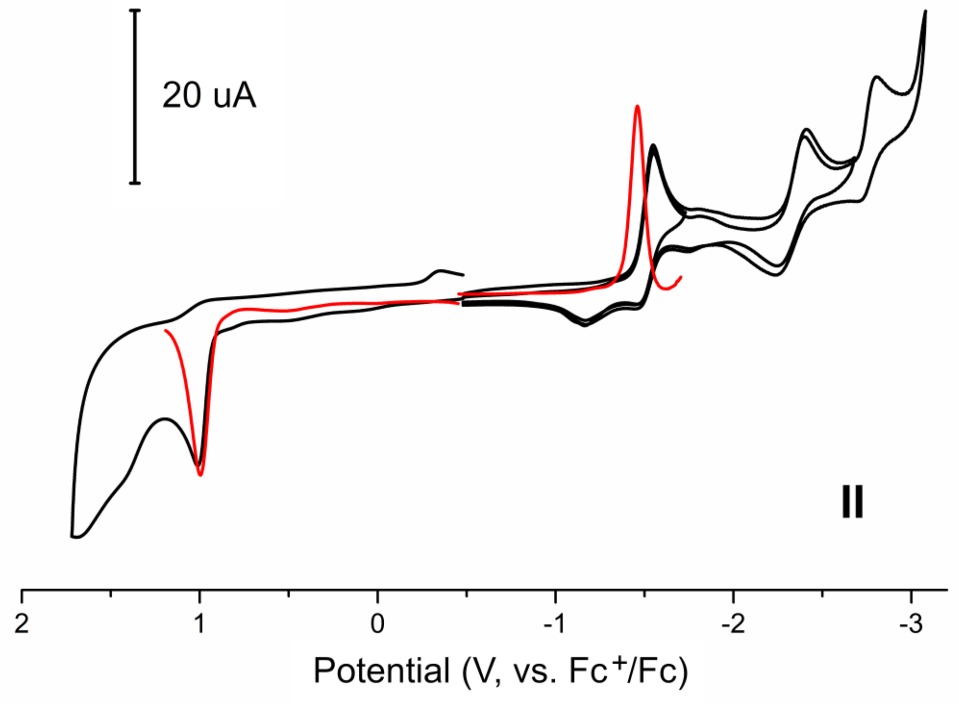
20 uA
II
2
1
0
-1
-2
-3
Potential (V, vs. Fc+/Fc)

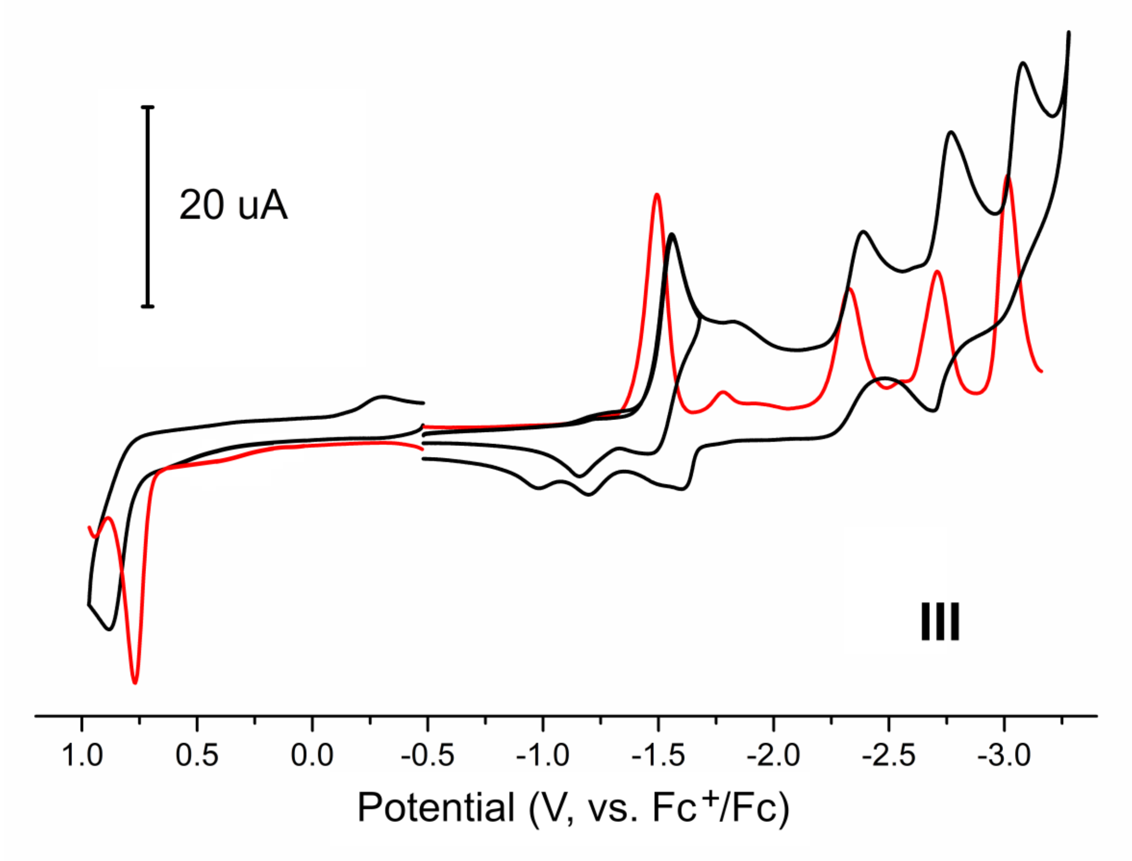
20 uA
III
1.0
0.5
0.0
-0.5
-1.0
-1.5
-2.0
-2.5
-3.0
Potential (V, vs. Fc⁺/Fc)

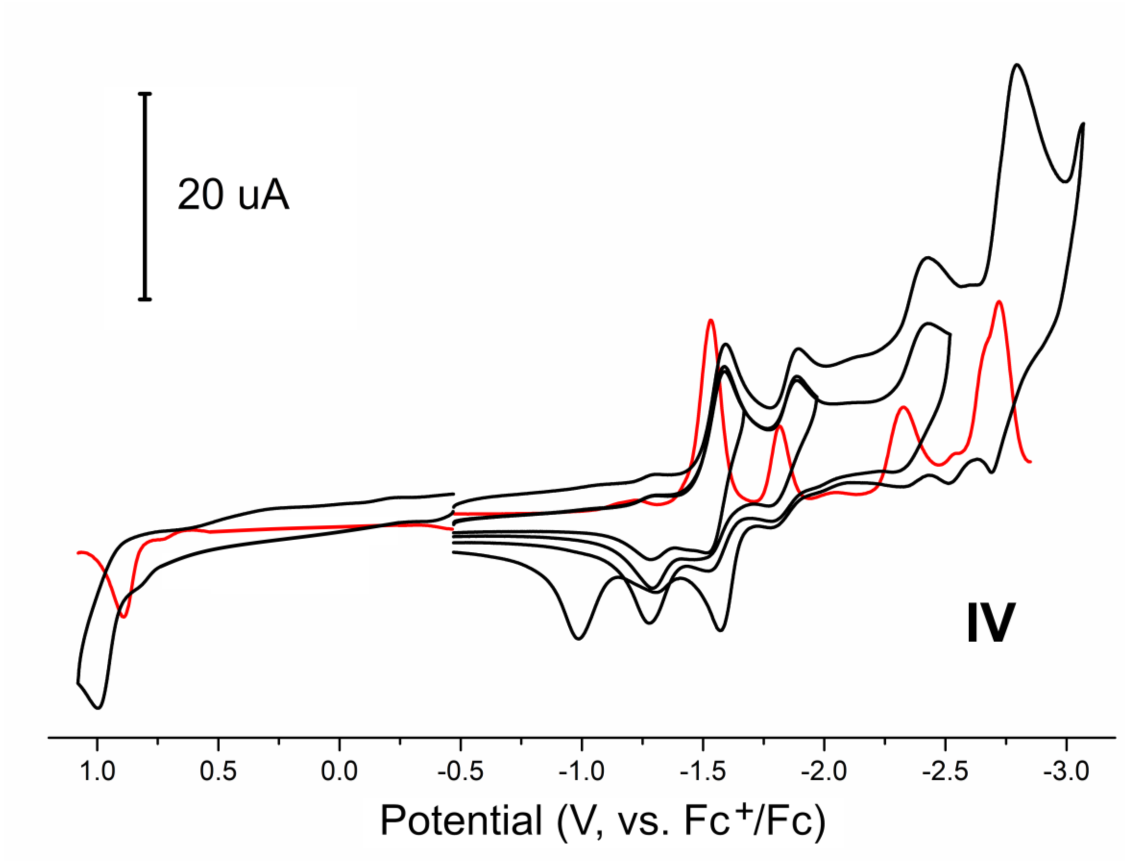
20 uA
IV
1.0
0.5
0.0
-0.5
-1.0
-1.5
-2.0
-2.5
-3.0
Potential (V, vs. Fc⁺/Fc)

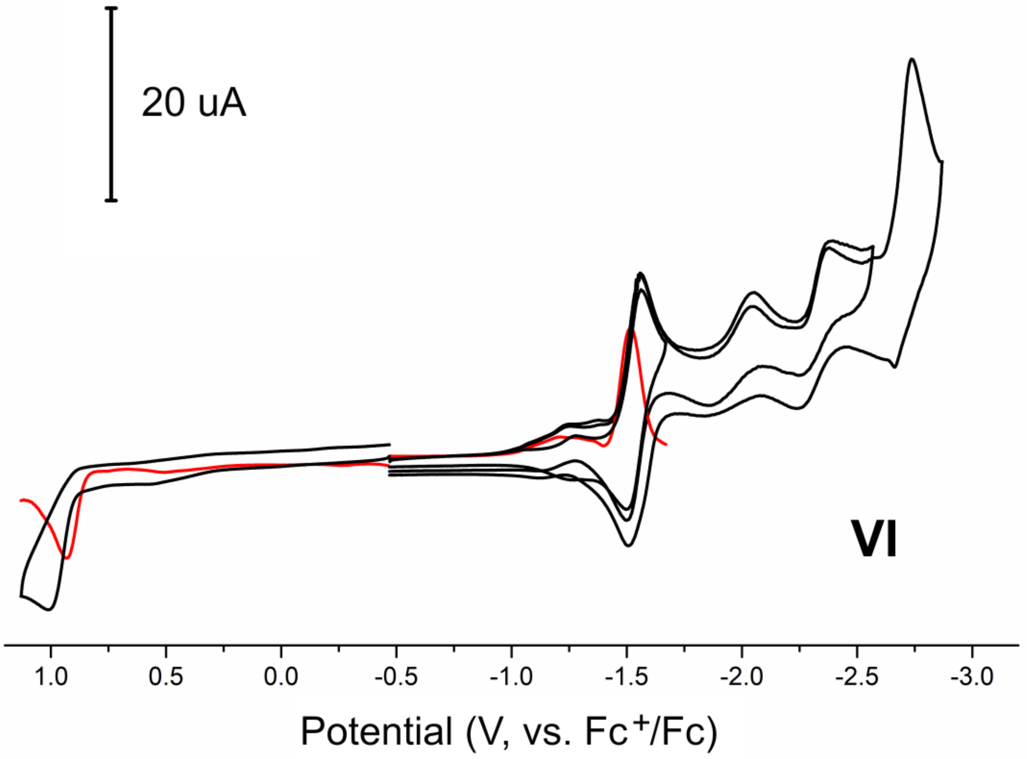
20 uA
VI
1.0
0.5
0.0
-0.5
-1.0
-1.5
-2.0
-2.5
-3.0
Potential (V, vs. Fc+/Fc)

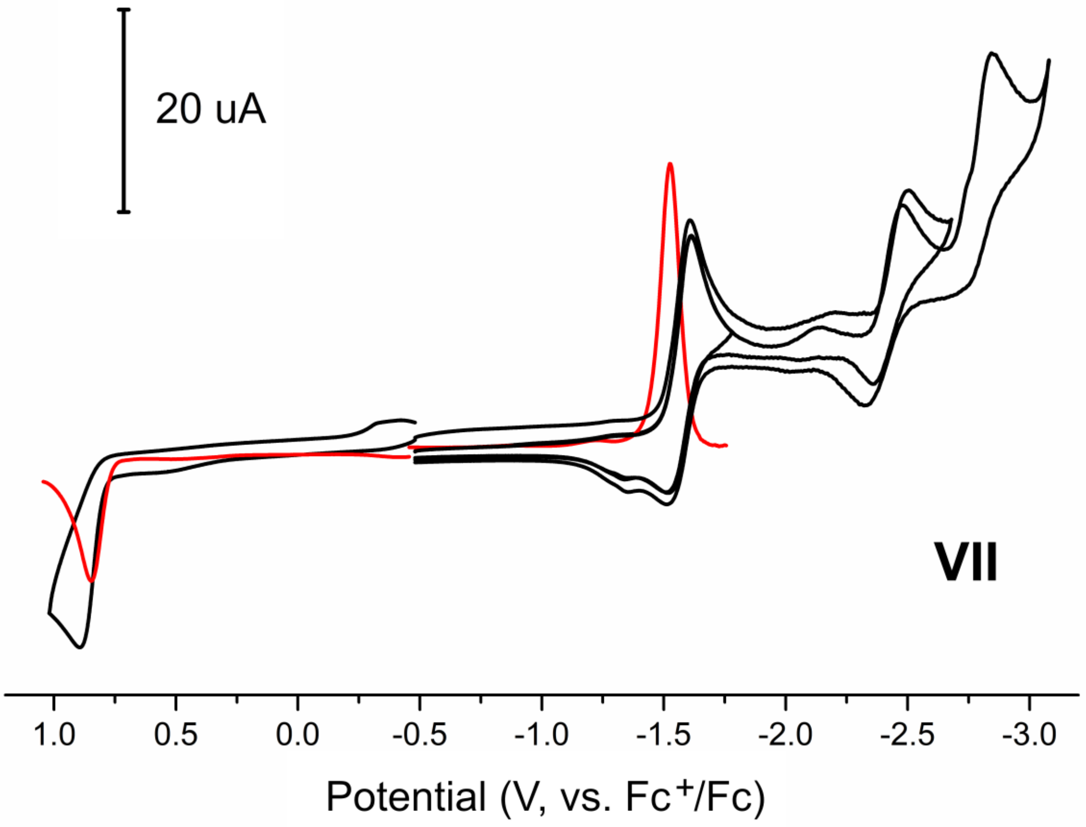
20 uA
VII
1.0
0.5
0.0
-0.5
-1.0
-1.5
-2.0
-2.5
-3.0
Potential (V, vs. Fc+/Fc)

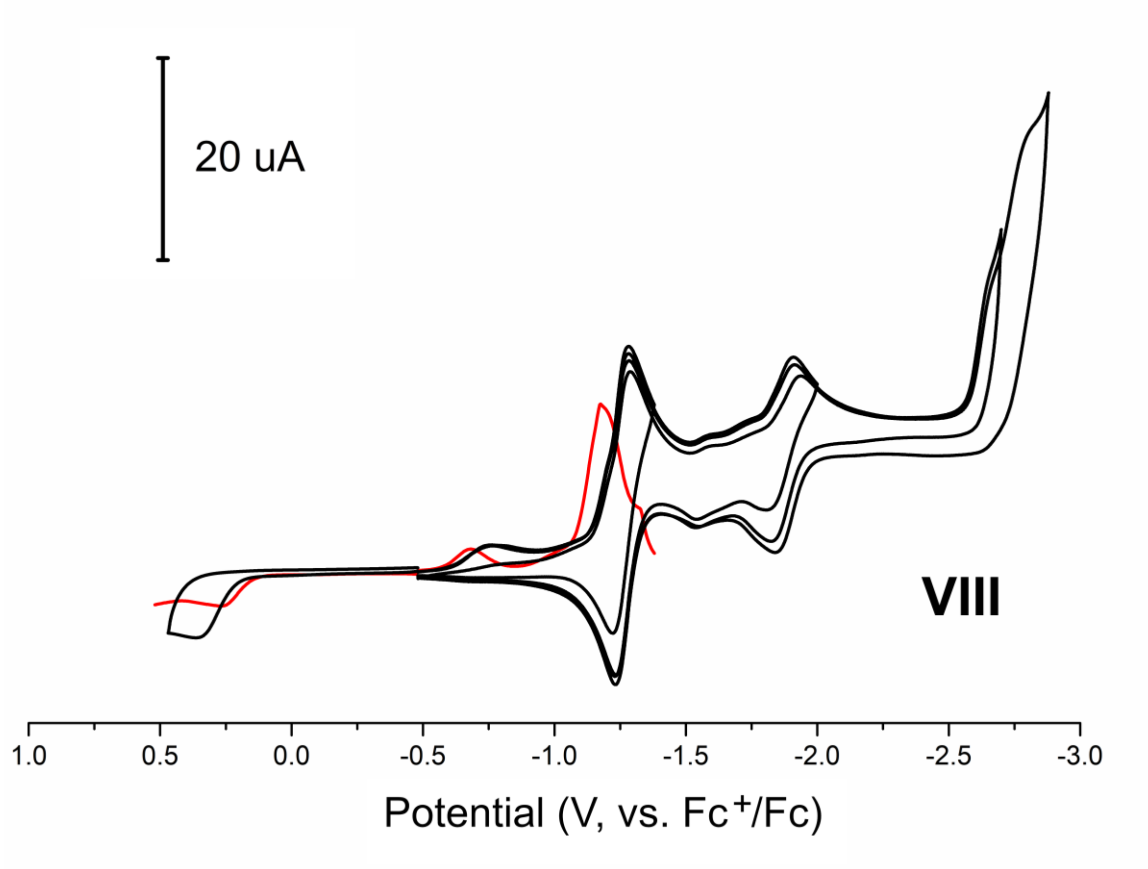

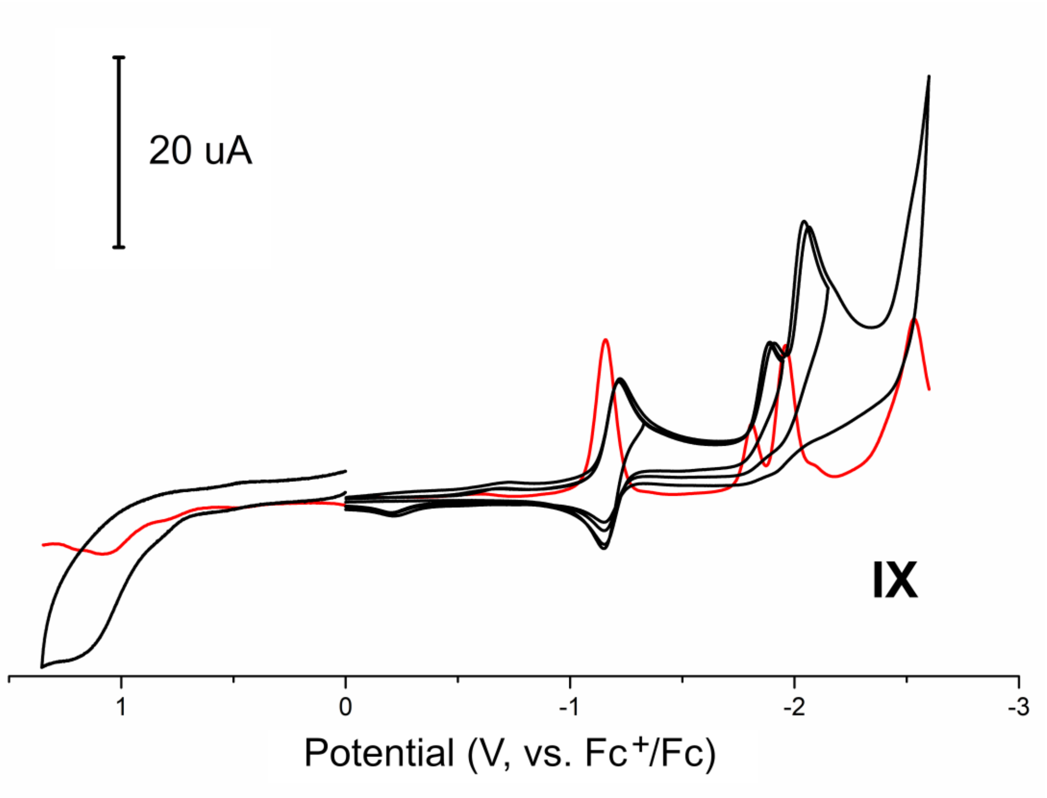

Figure S18. CVs and DPVs for **I-IX** in THF. Conditions: working electrode – glassy carbon, auxiliary electrode – Pt, supporting electrolyte – Bu_4NPF_6.

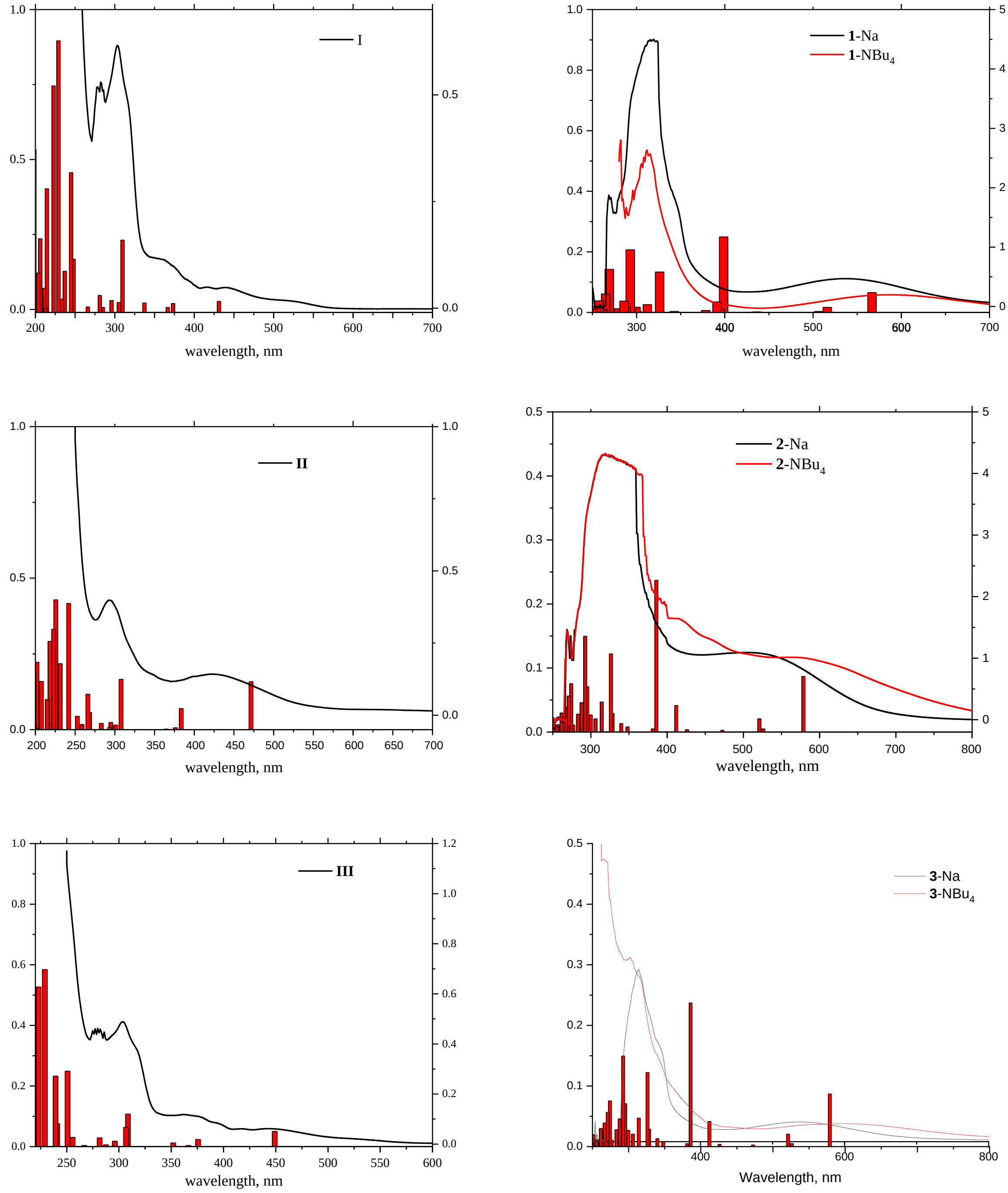
I
wavelength, nm
1-Na
1-NBu4
wavelength, nm
II
wavelength, nm
2-Na
2-NBu4
wavelength, nm
III
wavelength, nm
3-Na
3-NBu4
Wavelength, nm

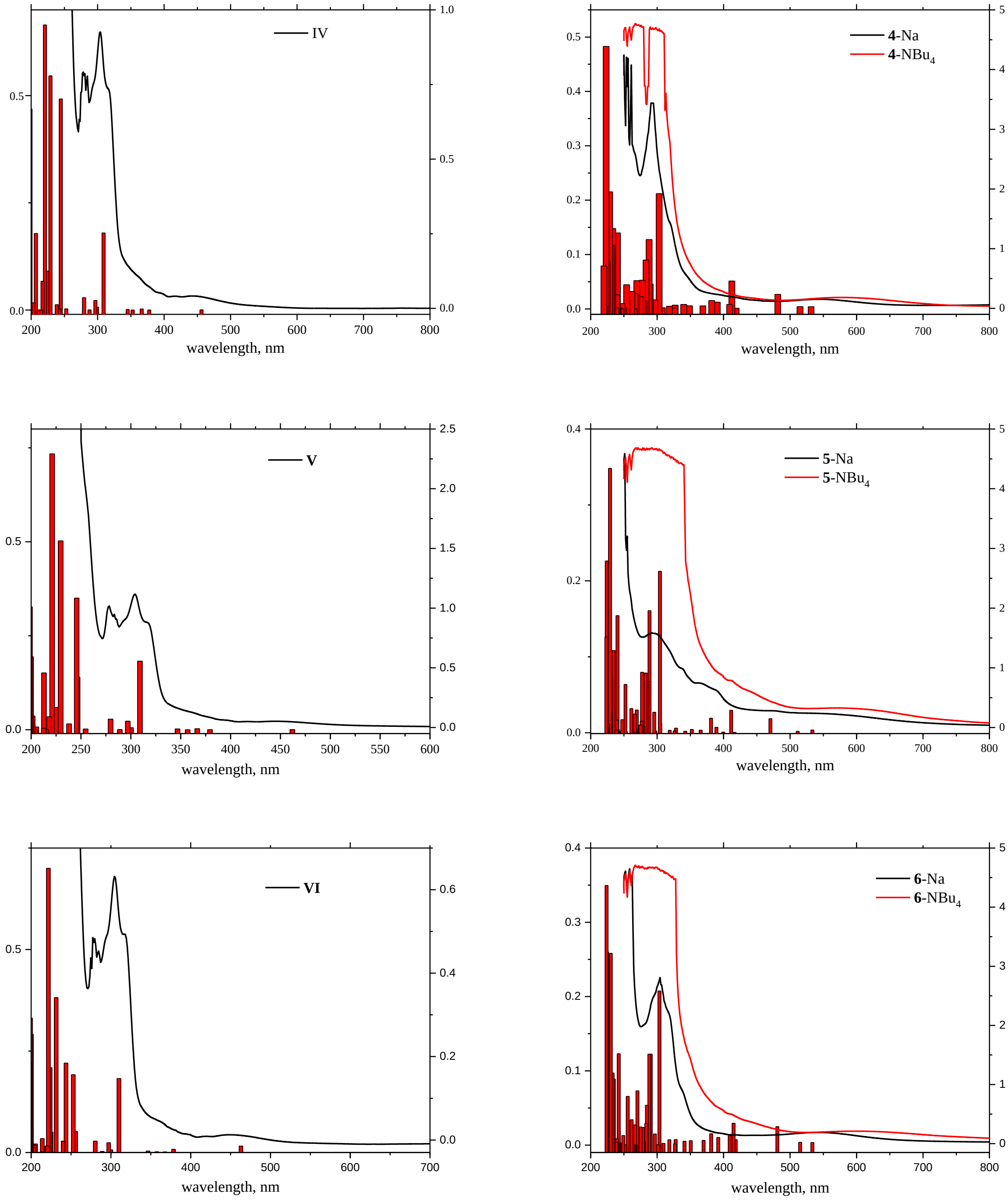

IV
wavelength, nm
4-Na
4-NBu4
wavelength, nm
V
wavelength, nm
5-Na
5-NBu4
wavelength, nm
VI
wavelength, nm
6-Na
6-NBu4
wavelength, nm

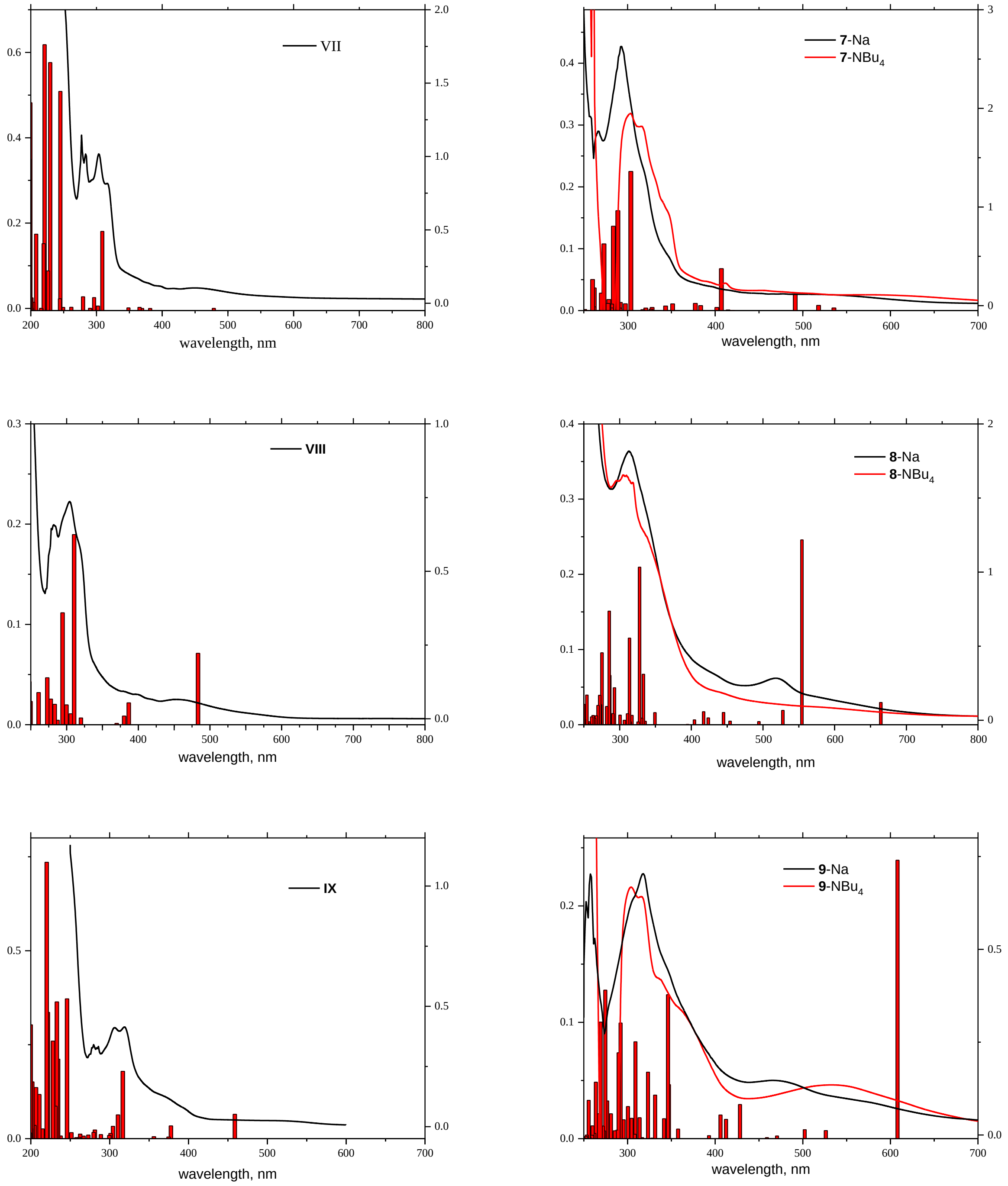

Figure S19. Experimental and predicted UV/Vis spectra for studied compounds.
